# The SpoIIQ‐SpoIIIAH complex of *C* *lostridium difficile* controls forespore engulfment and late stages of gene expression and spore morphogenesis

**DOI:** 10.1111/mmi.13311

**Published:** 2016-02-12

**Authors:** Mónica Serrano, Adam D. Crawshaw, Marcin Dembek, João M. Monteiro, Fátima C. Pereira, Mariana Gomes Pinho, Neil F. Fairweather, Paula S. Salgado, Adriano O. Henriques

**Affiliations:** ^1^ Microbial Development, Instituto de Tecnologia Química e Biológica, Universidade Nova de Lisboa, Avenida da República, Estação Agronómica Nacional, Avenida da República2780‐157OeirasPortugal; ^2^ Institute for Cell and Molecular Biosciences, Faculty of Medical Sciences, Newcastle UniversityNewcastle upon TyneUK; ^3^ MRC Centre for Molecular Bacteriology and Infection, Department of Life Sciences, Imperial College LondonLondonUK; ^4^ Bacterial Cell Biology Laboratory, Instituto de Tecnologia Química e Biológica, Universidade Nova de Lisboa, Avenida da República, Estação Agronómica Nacional, Avenida da República2780‐157OeirasPortugal; ^5^ Present address: University of Vienna, Department of Microbiology and Ecosystem Science, Division of Microbial EcologyAlthanstr. 141090ViennaAustria.

## Abstract

Engulfment of the forespore by the mother cell is a universal feature of endosporulation. In *Bacillus subtilis*, the forespore protein SpoIIQ and the mother cell protein SpoIIIAH form a channel, essential for endosporulation, through which the developing spore is nurtured. The two proteins also form a backup system for engulfment. Unlike in *B. subtilis*, SpoIIQ of *Clostridium difficile* has intact LytM zinc‐binding motifs. We show that *spoIIQ* or *spoIIIAH* deletion mutants of *C. difficile* result in anomalous engulfment, and that disruption of the SpoIIQ LytM domain via a single amino acid substitution (H120S) impairs engulfment differently. SpoIIQ and SpoIIQ^H120S^ interact with SpoIIIAH throughout engulfment. SpoIIQ, but not SpoIIQ^H120S^, binds Zn^2+^, and metal absence alters the SpoIIQ‐SpoIIIAH complex *in vitro*. Possibly, SpoIIQ^H120S^ supports normal engulfment in some cells but not a second function of the complex, required following engulfment completion. We show that cells of the *spoIIQ* or *spoIIIAH* mutants that complete engulfment are impaired in post‐engulfment, forespore and mother cell‐specific gene expression, suggesting a channel‐like function. Both engulfment and a channel‐like function may be ancestral functions of SpoIIQ‐SpoIIIAH while the requirement for engulfment was alleviated through the emergence of redundant mechanisms in *B. subtilis* and related organisms.

## Introduction

Intercellular communication, crucial for the coordinated behavior of cells, often involves gap junctional protein channels that span the lipid bilayers of adjacent cells allowing them to exchange ions and small water‐soluble molecules to maintain cellular homeostasis. These intercellular connections are formed by oligomers of membrane‐anchored channel‐forming protein subunits, with each of the two contacting cells contributing half of the channel (Houghton, 2005). Gap‐junctions are found in virtually all animal cells, and resemble analogous structures found in plant cells, called plasmodesmata (Brunkard *et al*., 2015). Gap‐junction‐mediated transport can, for example, allow metabolic cooperation, whereby a cell transfers nutrients or intermediate metabolites to an adjacent cell, itself unable to synthesize or acquire them. A membrane protein complex formed during endospore development by the bacterium *Bacillus subtilis*, is used by the mother cell as a conduit to nurture the developing endospore in a gap junction‐type function (Blaylock *et al*., 2004; Camp and Losick, 2008; Meisner *et al*., 2008; Doan *et al*., 2009).

**Figure 1:**
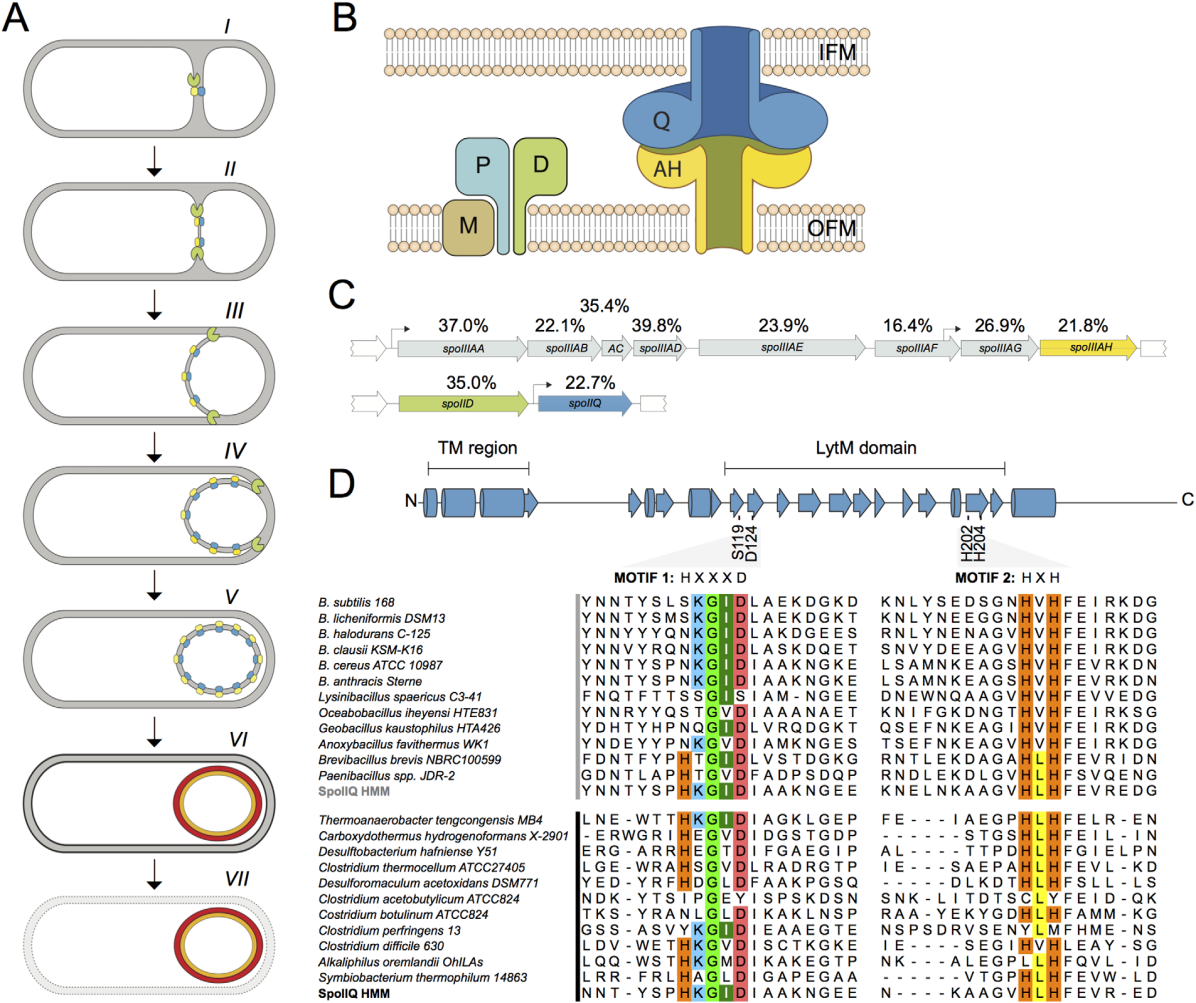
Sporulation and the SpoIIQ‐SpoIIIAH channel. A. Schematic representation of the main stages of sporulation. Asymmetric cell division at one of the poles gives rise to the small daughter cell or forespore, side‐by‐side with the mother cell (*I*). As sporulation progresses, the mother cell engulfs the forespore within its cytoplasm. In *B. subtilis*, the DMP protein complex (green) is essential for hydrolysis of PG within the septum during the initial stages of engulfment (*II‐IV*) (see text for details; see also panel B). In *B. subtilis* and presumably other sporeformers, SpoIIQ (blue) and SpoIIIAH (yellow), together with additional *spoIIIA‐*encoded proteins (not represented), are also involved in engulfment and required for sporulation (*III‐V*). Spore maturation involves the formation of two main protective layers (the cortex PG and the coats) in the intermembrane space (*VI*). It is not known whether the SpoIIQ‐SpoIIIAH channel is maintained at these late stages in Clostridia and other endosporeformers, but the two proteins are degraded following engulfment completion in *B. subtilis* (Jiang *et al*., 2005; Meisner *et al*., 2008). Finally, the mother cell lyses, releasing the mature spore into the media (*VII*). B. SpoIIQ‐SpoIIIAH and DMP organization. The proposed organization in *B. subtilis*, of SpoIIQ (blue) and SpoIIIAH (yellow) multimeric rings in the inner (IFM) and outer (OFM) forespore membranes is shown schematically. The interaction of the two rings is proposed to form a channel that allows communication between the mother cell and the forespore. The DMP complex (SpoIID, D in green; SpoIIP, P in light blue; and SpoIIM, M, in brown) required for PG remodeling during engulfment in *B. subtilis* is also represented (see text for details). C. Schematic representation of the octacistronic *spoIIIA* (top) and *spoIID*‐*spoIIQ* operons (bottom) of *C. difficile*. The position of two σ^E^‐dependent promoters identified in the *spoIIIA* operon of *B. subtilis* is shown by bent arrows. The figure also shows the percentage of sequence similarity between the indicated proteins of *C. difficile* 630Δ*erm* and their counterparts in *B. subtilis*. D. LytM motifs in Firmicutes. *B. subtilis* SpoIIQ, with the LytM domain and the position of the conserved residues in motifs 1 and 2 indicated, with the secondary structure elements observed in the structural models labelled (Meisner *et al*., 2012; Levdikov *et al*., 2012) (cylinders: α helices; arrows:β strands). A sequence alignment of SpoIIQ orthologues found in representative *Bacilli* (top group) and *Clostridia* (bottom group) (Galperin *et al*., 2012) when compared to the Hidden Markov Model sequence (HMM) previously described (Crawshaw *et al*., 2014) shows the relative conservation of the LytM catalytic residues (HxxxD, motif 1 and HxH, motif 2, highlighted). Sequences were retrieved and the HMM sequence created as previously described (Crawshaw *et al*., 2014). Colors indicate conservation when compared to the HMM sequence.

Endospore differentiation in *B. subtilis* involves two cells formed at the onset of the process through polar division of the rod‐shaped cell (Fig. 1A). The smaller cell, or forespore, will become the future spore, while the larger mother cell nurtures spore development but ultimately lyses to release the mature spore into the environment (Hilbert and Piggot, 2004; Stragier and Losick, 1996). At the time of division, the two cells are in direct contact with the external medium (Fig. 1A). However, soon after division, the mother cell membrane begins to engulf the forespore, eventually transforming it into a free protoplast surrounded by two membranes of opposing polarity. Engulfment completion marks the transition to the last stages in development, during which spore morphogenesis is completed and the spore is prepared for dormancy (Fig. 1A). The forespore and the mother cell follow different programs of gene expression, largely defined by a cascade of cell type‐specific RNA polymerase sigma (σ) factors, but cell‐cell communication pathways coordinate and keep the two programs in harmony with the sequence of morphogenesis (Losick and Stragier, 1992; Stragier and Losick, 1996; Kroos and Yu, 2000; Rudner and Losick, 2001). σ^F^ is activated in the forespore soon after polar division and it controls early stages of development in this cell. σ^F^ is also responsible for the activation of the early mother cell‐specific regulatory protein σ^E^. The onset of σ^G^ activity coincides with engulfment completion, and leads to activation of σ^K^, which replaces σ^E^ in the mother cell. Moreover, σ^G^ activity requires expression of the σ^F^‐controlled forespore gene *spoIIQ* (Londono‐Vallejo *et al*., 1997) and of the mother cell σ^E^‐controlled *spoIIIA* operon (Illing and Errington, 1991b) (Fig. 1B). In the absence of any of the *spoIIIA‐*encoded proteins or SpoIIQ the forespore collapses and gene expression in the forespore halts (Serrano *et al*., 2004; Doan *et al*., 2005; 2009; Camp and Losick, 2008, 2009). SpoIIIAH and SpoIIQ localize to the asymmetric septum and to the engulfing membranes, and the two proteins interact in the intermembrane space *via* their extracytoplasmic domains (Rubio and Pogliano, 2004; Blaylock *et al*., 2004; Doan *et al*., 2005; 2009). This interaction localizes SpoIIIAH to the septum and to the engulfing membranes but the primary tether for SpoIIQ, although dependent on mother cell gene expression, is still unclear (Blaylock *et al*., 2004; Rubio and Pogliano, 2004; Fredlund *et al*., 2013; Rodrigues *et al*., 2013). SpoIIQ and SpoIIIAH are thought to form a scaffold for the assembly of a larger complex that includes the remaining *spoIIIA‐*encoded proteins (Chiba *et al*., 2007; Doan *et al*., 2009). Evidence suggests that SpoIIIAH and SpoIIQ form a direct channel between the cytoplasm of the mother cell and the forespore (Meisner *et al*., 2008). SpoIIIAH is related to the YcsJ/FliF family of proteins that form oligomeric rings in Type III secretion systems and the flagellar basal body, and the structure of a SpoIIQ‐SpoIIIAH co‐crystal confirms the potential of the complex to form two apposed ring‐like structures (Meisner *et al*., 2012; Levdikov *et al*., 2012). Importantly, the C‐terminus of SpoIIIAH can be labelled by a forespore‐produced heterologous biotin ligase in a SpoIIQ‐dependent manner, providing direct evidence for a channel (Meisner *et al*., 2008). The strong similarity of SpoIIIAA to transport ATPases, and the varying degrees of similarity of the remaining SpoIIIA proteins with components of Type II/IV secretion systems, together with the phenotypes caused by mutations in the ATP binding motifs of SpoIIIAA, suggest a specialized system for transport into the forespore (Blaylock *et al*., 2004; Camp and Losick, 2008; Meisner *et al*., 2008; Doan *et al*., 2009). In *B. subtilis spoIIQ* or *spoIIIAH* mutants, the activity of σ^G^ and the continued activity of σ^F^, as well as the activity of the heterologous single chain RNA polymerase from phage T7, produced from a σ^F^ dependent promoter, is severely curtailed (Camp and Losick, 2009). This led to a model in which the SpoIIQ‐SpoIIIAH complex functions as a gap‐junction‐like feeding tube, through which the mother cell supplies the forespore with small molecules required to maintain its metabolic potential following insulation from the external medium (Camp and Losick, 2009; Doan *et al*., 2009). The molecules conveyed to the forespore by the SpoIIQ‐SpoIIIAH complex remain unknown. Isolation of the forespore upon engulfment completion is a hallmark of endosporulation. Accordingly, the *spoIIIA* operon and *spoIIQ* are conserved and part of a signature for endosporulation (Galperin *et al*., 2012; Abecasis *et al*., 2013; Crawshaw *et al*., 2014) (Fig. 1C). The prediction that the function of *spoIIIA* and *spoIIQ* is conserved has not been tested.

Engulfment is mainly controlled by a complex of three proteins produced under the control of σ^E^ (Abanes‐De Mello *et al*., 2002; Chastanet and Losick, 2007; Aung *et al*., 2007; Gutierrez *et al*., 2010; Morlot *et al*., 2010) (Fig. 1A and B). Within this complex, termed the DMP machine, SpoIID and SpoIIP are peptidoglycan (PG) hydrolases that act coordinately to generate the main force for advancing the leading edge of the mother cell membrane (Abanes‐De Mello *et al*., 2002; Aung *et al*., 2007; Chastanet and Losick, 2007; Morlot *et al*., 2010; Gutierrez *et al*., 2010). *spoIID*, *spoIIM* or *spoIIP* mutants are blocked in the initial stage of engulfment, septal PG thinning (Lopez‐Diaz *et al*., 1986; Smith *et al*., 1993; Bylund *et al*., 1994; Frandsen and Stragier, 1995; Illing and Errington, 1991a; Aung *et al*., 2007) (Fig. 1A). However, when the activity of the DMP machine is compromised, two redundant processes gain importance. One is synthesis of PG at the leading edges of the engulfing membranes (Meyer *et al*., 2010). The second is a backup mechanism provided by the strong SpoIIQ‐SpoIIIAH interaction across the intermembrane space. Importantly, the SpoIIQ‐SpoIIIAH interaction is also capable of driving engulfment independently of the DMP machine in protoplasts generated by enzymatic removal of the PG (Broder and Pogliano, 2006). The SpoIIQ‐SpoIIIAH interaction may facilitate engulfment by a Brownian ratchet‐like mechanism, in which thermal fluctuations of the engulfing membrane create new sites of SpoIIQ‐SpoIIIAH interaction that prevent membrane retraction (Broder and Pogliano, 2006; Meyer *et al*., 2010; Gutierrez *et al*., 2010; Rodrigues *et al*., 2013; Ojkic *et al*., 2014). While the essential function of the two proteins during *B. subtilis* sporulation appears to be in the assembly of the feeding channel, several observations suggest that SpoIIQ‐SpoIIIAH may in fact represent an ancestral mechanism for engulfment, masked by redundancy (Ojkic *et al*., 2014). Firstly, the similarity of the C‐terminal domain of SpoIIIAH to the YscJ family suggests an old origin for the protein (Ojkic *et al*., 2014). Secondly, SpoIIQ has a LytM endopeptidase domain found in several cell wall hydrolases (Ramadurai and Jayaswal, 1997; Firczuk *et al*., 2005; Camp and Losick, 2008; Meisner *et al*., 2008; Rodrigues *et al*., 2013). While in *B. subtilis* and most *Bacilli*, this domain lacks a critical histidine residue required for Zn^2+^ binding and the formation of a catalytic center, in several clostridial species the domain is intact, suggesting that it could contribute directly to PG degradation during engulfment (Crawshaw *et al*., 2014) (Fig. 1D). It is also interesting that while SpoIIIAH and SpoIIQ interact *via* their YscJ and LytM domains respectively, a second surface in the LytM domain may be involved in an interaction with another protein(s), possibly the DMP machine or the products of its activity. This interaction seems to maintain the localization of SpoIIQ even in the absence of SpoIIIAH (Rodrigues *et al*., 2013; Fredlund *et al*., 2013). Finally, under certain nutritional conditions, SpoIIQ and SpoIIIAH are required for engulfment, even in the presence of the DMP machine, suggesting that SpoIIQ and SpoIIIAH could have more important roles in engulfment in other organisms (Sun *et al*., 2000; Broder and Pogliano, 2006; Aung *et al*., 2007).

Recent improvements in the available genetic tools have spurred studies of spore development in clostridial species, including *C. difficile*, a major nosocomial pathogen (Fimlaid *et al*., 2013; Pereira et al., 2013; Saujet *et al*., 2013). In *C. difficile*, although the main stages of sporulation and the principal functions of the cell type‐specific sigma factors appear largely maintained, the cell‐cell communication pathways are less conserved. Importantly, the activity of σ^G^ was shown to be partially independent of σ^E^ (Saujet *et al*., 2013; Pereira *et al*., 2013; Fimlaid *et al*., 2013). Together with the presence of an intact LytM domain in the *C. difficile* orthologue of SpoIIQ, this raised questions about the function of SpoIIQ‐SpoIIIAH in this organism.

Here, we have analyzed the function of the *spoIIQ* and *spoIIIAH* genes in *C. difficile*, with hopes that novel insights into the function of the SpoIIQ‐SpoIIIAH complex would emerge. We show that, in *C. difficile*, SpoIIQ and SpoIIIAH are required for engulfment, but also for late stages in spore morphogenesis. We also show that an intact LytM domain in SpoIIQ is at least partially dispensable for normal engulfment. Our study shows that SpoIIQ and SpoIIIAH interact during engulfment and form a complex *in vitro*. We establish that SpoIIQ binds Zn^2+^, and absence of this metal alters the SpoIIQ‐SpoIIIAH interaction. *spoIIQ* or *spoIIIAH* mutants are impaired in late gene expression and we propose that Zn^2+^‐binding by SpoIIQ facilitates a late channel‐like function of the complex. Evidence is presented that the activity of SpoIIQ‐SpoIIIAH is required for late transcription in both sporangial chambers. Our study also suggests that both control of engulfment and a channel‐like function are ancestral functions of the SpoIIQ‐SpoIIIAH complex.

## Results

### *spoIIQ* and *spoIIIAH* in‐frame deletion mutants are blocked in sporulation

**Figure 2:**
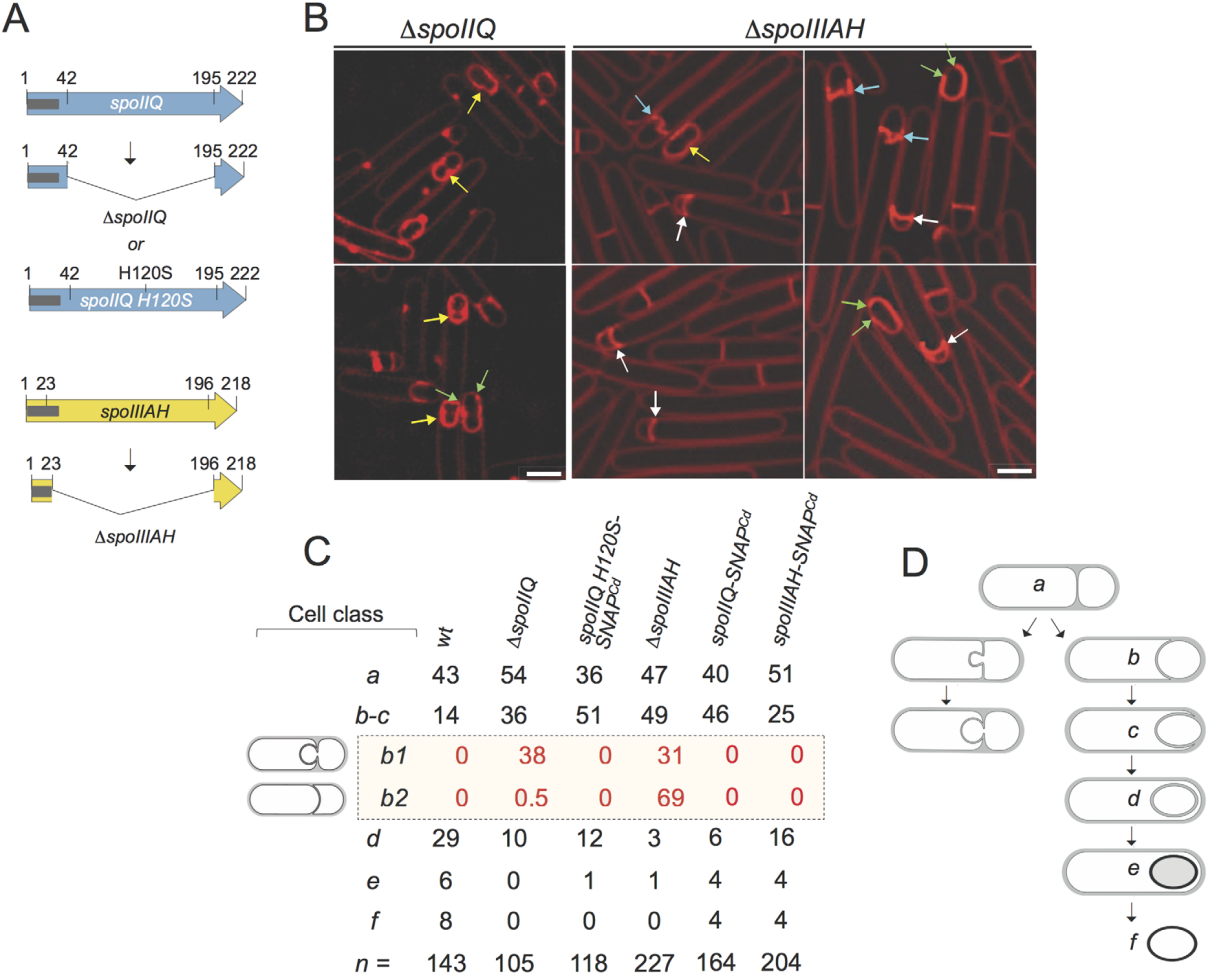
*spoIIQ* and *spoIIIAH* mutants of *C. difficile* are blocked early in the engulfment sequence. A. Schematic representation of the various alleles used. The Δ*spoIIQ* deletion removed codons 42 through 195 of the 222‐codons‐long *spoIIQ* gene, whereas in Δ*spoIIIA* codons 23–196 of the 218‐codons‐long coding region were deleted. Both are in‐frame deletion mutations, generated by allele‐coupled exchange (ACE); the s*poIIQ H120S‐SNAP^Cd^* allele is expressed from a plasmid; note that the *SNAP^Cd^* moiety is not represented for simplicity. The grey lines represent the region coding for the transmembrane segments of SpoIIQ or SpoIIIAH. B. SR‐SIM images of *spoIIQ* and *spoIIIAH* sporangia. The cells were collected from 14 hours SM cultures and stained with FM4‐64. The yellow arrows point to sporangia with bulges or vesicles; the blue arrows point to septa in which bulges form off center; the white arrows indicate septa that curve into the forespore, and the green arrows show cases of asymmetric migration of the engulfing membranes. Scale bar, 2 µm. C. Scoring of the percentage of cells in each of the morphological classes of sporulation (*a* to *f*), as seen by phase contrast and fluorescence microscopy (see text for details) for the indicated strains after 14 hours of growth in SM. The percentage of cells in each class is relative to the number of sporulating cells; ‘n’ is the total number of cells scored. Note that class *b‐c* was divided into sub‐classes *b1* (bulges, vesicles and asymmetrically migrating membranes) and *b2* (septa curving into the forespore); for sub‐classes *b1* and *b2*, the numbers indicate the percentage of the cells scored as class *b‐c* showing the indicated phenotypes. D. Schematic representation of the engulfment pathway in WT cells (right) and in DMP mutants of *B. subtilis* (left).

A recent study used mutagenesis with a *mariner* transposon followed by transposon‐directed insertion site sequencing (TraDIS) to identify a set of 798 genes whose disruption reduced the efficiency of sporulation by *C. difficile* (Dembek *et al*., 2015). Among the genes identified and studied in more detail was the orthologue (CD630_01250) of the *B. subtilis spoIIQ* gene. An in‐frame deletion mutant of *spoIIQ*, lacking codons 42–195, was generated by allele‐coupled exchange, or ACE (Fig. 2A) in the background of the widely used 630Δ*erm* strain, modified to bear a truncated *pyrE* gene (Ng *et al*., 2013). The Δ*spoIIQ* mutant (*spoIIQ* for simplicity) was shown to initiate sporulation in a rich medium (Brain Heart Infusion Supplement, BHIS) but to be severely impaired in heat resistant spore formation; however, the stage of block was not examined in detail (Dembek *et al*., 2015). Introduction of the wild type (WT) *spoIIQ* gene at the *pyrE* locus restored sporulation (Dembek *et al*., 2015). Here, we used ACE to similarly construct a *spoIIIAH* allele, in which codons 23–196 were removed in frame (Fig. 2A), and to transfer the mutation to the *spoIIIAH* locus of strain 630Δ*erm*. The steps involved in the construction of the Δ*spoIIIAH* mutant, hereinafter referred to as *spoIIIAH* for simplicity (strain AHCD812; Supporting Information Table S1), are outlined in Supporting Information Fig. S1 (see also the Supporting Experimental procedures). To facilitate the identification of the stage of block imposed by the *spoIIQ* or *spoIIIAH* mutations, as well as their impact on cell type‐specific gene expression at the single cell level (see below), we used liquid sporulation medium (SM), in which we found sporulation to be more synchronized than in other growth conditions tested (Pereira *et al*., 2013). The *spoIIQ* mutant (Dembek *et al*., 2015) and the newly constructed *spoIIIAH* mutant, along with the parental WT strain 630Δ*erm* were grown in SM, and the titer of heat resistant spores (spores/ml of culture) was determined 24, 48 and 72 h after inoculation. In line with earlier results (Pereira *et al*., 2013), the titer of spores for the WT strain increased from 1.7 × 10^5^ ± 2.9 × 10^4^ spores/ml of culture at hour 24, to 1.5 × 10^6^ ± 4.5 × 10^5^ spores/ml at hour 48 and 2.1 × 10^6^ ± 3.7 × 10^5^ spores/ml at hour 72 (Table 1). In contrast, while the *spoIIQ* or *spoIIIAH* mutations did not affect cell viability, no heat resistant spores were detected for either mutant, at any sampling time (Table 1). Thus, the *spoIIQ* mutation had a more severe impact on the titer of heat resistant spores in SM, as compared to BHIS (Dembek *et al*., 2015). In any event, under our culturing conditions, *spoIIQ* and *spoIIIAH* are essential for heat resistant spore formation.

**Table 1:** Total and heat resistant (Heat^R^) cell counts (CFU/ml) for the indicated strains grown in SM.

Time (h)^a^	630Δ*erm*		*spoIIQ*		*spoIIIAH*		*spoIIQ‐SNAP^Cd^*		*spoIIIAH‐SNAP^Cd^*		*spoIIQ H120S‐SNAP^Cd^*	
	Total^b^	Heat^R^	Total	Heat^R^	Total	Heat^R^	Total	Heat^R^	Total	Heat^R^	Total	Heat^R^
24	9.2 × 10^7^ ± 7.6 × 10^6^	1.7 × 10^5^ ± 2.9 × 10^4^	8.2 × 10^7^ ± 6.7 × 10^6^	0	1.1 × 10^8^ ± 1.8 × 10^7^	0	7.6 × 10^7^ ± 3.8 × 10^6^	4.8 × 10^5^ ± 1.3 × 10^4^	6.4x10^7^ ± 3.1 × 10^6^	1.2 × 10^5^ ± 2.9 × 10^4^	1.3 × 10^8^ ± 4.9 × 10^7^	4.5 × 10^3^ ± 5.0 × 10^2^
48	2.5 × 10^7^ ± 4.3 × 10^6^	1.5 × 10^6^ ± 4.5 × 10^5^	1.0 × 10^7^ ± 1.0 × 10^6^	0	3.2 × 10^7^ ± 2.0 × 10^6^	0	3.4 × 10^7^ ± 3.3 × 10^6^	4.7 × 10^5^ ± 1.6 × 10^4^	3.5x10^7^ ± 2.6 × 10^6^	1.5 × 10^6^ ± 2.8 × 10^5^	2.2 × 10^7^ ± 5.0 × 10^5^	5.3 × 10^3^ ± 4.5 × 10^2^
72	3.4 × 10^7^ ± 7.1 × 10^6^	2.1 × 10^6^ ± 3.7 × 10^5^	2.9 × 10^7^ ± 4.3 × 10^6^	0	2.6 × 10^7^ ± 3.3 × 10^6^	0	3.9 × 10^7^ ± 3.5 × 10^6^	2.9 × 10^5^ ± 4.4 × 10^4^	3.8 × 10^7^ ± 4.0 × 10^6^	1.9 × 10^6^ ± 1.0 × 10^5^	3.6 × 10^7^± 2.3 × 10^6^	7.83 × 10 ± 2.4 × 10^2^

a Time in hours after inoculation into liquid sporulation medium (sm).

b Values represent the average ± standard deviation (SD) of three independent experiments.

Even though the *spoIIIAH* mutation is an in‐frame deletion, we tested for polar effects by *in trans* complementation analysis. We constructed a plasmid in which *spoIIIAH*, expressed from the promoter for the *spoIIIA* operon, was fused at its 3′‐end to the *SNAP^Cd^* reporter (Pereira *et al*., 2013). In parallel, a similar plasmid was produced bearing a *spoIIQ‐SNAP^Cd^* fusion expressed from the *spoIIQ* promoter. The ability of the *spoIIQ*‐*SNAP^Cd^* or *spoIIIAH*‐*SNAP^Cd^* alleles to complement the *spoIIQ* or *spoIIIAH* mutations was assessed by measuring the titer of heat resistant spores at hours 24, 48 and 72 of growth in SM. The *spoIIQ* mutant complemented with the *spoIIQ‐SNAP^Cd^* allele gave a titer of 4.8 × 10^5^ ± 1.3 × 10^4^ spores/ml of culture at hour 24 (as compared with 1.7 × 10^5^ ± 2.9 × 10^4^ spores/ml for the WT), 4.7 × 10^5^ ± 1.6 × 10^4^ at hour 48 (1.5 × 10^6^ ± 4.5 × 10^5^ for the WT) and 2.9 × 10^5^ ± 4.4 × 10^4^ at hour 72 (2.1 × 10^6^ ± 3.7 × 10^5^ for the WT) (Table 1). Although largely restoring sporulation to the *spoIIQ* mutant, the *spoIIQ‐SNAP^Cd^* allele seemed to perform better at early time points. The *spoIIIAH* mutant complemented with the fusion showed titers of 1.2 × 10^5^ ± 2.9 × 10^4^ spores/ml of culture at hour 24, 1.5 × 10^6^ ± 2.8 × 10^5^ at hour 48, and 1.9 × 10^6^ ± 1.0 × 10^5^ at hour 72 of growth in SM (Table 1). Thus, *spoIIIAH‐SNAP^Cd^* fully restored sporulation to the *spoIIIAH* mutant. We conclude that the sporulation phenotype of the two mutants is thus due to loss of function of the *spoIIQ* or *spoIIIAH* genes (see also (Dembek *et al*., 2015)). Together, the results confirm the requirement for sporulation of the *spoIIQ* and *spoIIIAH* genes, suggest that the impact of the *spoIIQ* mutation may be medium‐dependent as also found for *B. subtilis* (Sun *et al*., 2000; Broder and Pogliano, 2006; Aung *et al*., 2007), and show that the SpoIIQ‐SNAP^Cd^ and SpoIIIAH*‐*SNAP^Cd^ fusions are largely functional.

### *spoIIQ* and *spoIIIAH* mutants are often blocked at an intermediate stage in the engulfment sequence

To determine the stage at which the *spoIIQ* and *spoIIIAH* mutations caused a defect in the spore differentiation pathway, cultures of the two mutants, in parallel with the parental strain, were grown in SM and imaged after 14 hours of growth, following staining with the membrane dye FM4‐64 and with the DNA marker Hoechst 33342. Previous work has shown that at this time of incubation in SM medium, all of the main stages of sporulation, including free mature (phase bright) spores, are represented in a culture of the WT (Pereira *et al*., 2013); therefore these conditions were used throughout this study, unless otherwise stated. The cells were imaged using Super Resolution Structured Illumination Microscopy (SR‐SIM) in which the lateral resolution is increased to about 110 nm, as compared with the diffraction limit of about 250 nm of conventional light microscopy (Schermelleh *et al*., 2010). As described previously (Pereira *et al*., 2013), the forespore chromosome stains strongly following asymmetric division and engulfment, while the more dispersed mother cell chromosome shows a less intense signal. The *spoIIQ* and *spoIIIAH* mutants completed asymmetric division and showed strong staining of the forespore chromosome (Supporting Information Fig. S2, blue and red arrows; compare A with B and C). Thus, neither asymmetric division nor segregation and condensation of the forespore chromosome are blocked in *spoIIQ* or *spoIIIAH* sporangia. Asymmetric division and forespore chromosome segregation were also observed for the *spoIIQ* mutant in BHIS medium (Dembek *et al*., 2015). We then scored cells based on morphological classes that can be visualized by phase contrast and fluorescence microscopy (Fig. 2B and C). To better visualize the asymmetric septum and the forespore membranes during engulfment in *spoIIQ* and *spoIIIAH* sporangia, the cells were imaged by SR‐SIM following FM4‐64 staining, but omitting the DNA labelling step (Fig. 2B). Class *a*, defined as cells with flat septa, was scored individually as was class *d*, defined as completed engulfment. However, cells with curved septa, i.e. at intermediate stages of engulfment were scored together as class *b‐c*. About 43% of the sporulating cells of the WT strain 630Δ*erm* showed straight, flat, septa (class *a*), 14% showed curved septa (class *b‐c*), and about 43% of the cells had completed the engulfment sequence (sum of classes *d* to *f*) (Fig. 2C); 6% showed discernible spores inside the mother cell (class *e*; which comprises sporangia with phase dark, phase grey or phase bright spores), and free spores represented 8% of the population of sporulating cells (class *f*) (Fig. 2C). In contrast, no cells scored as class *e* or *f* for the *spoIIQ* mutant and only 1% of the cells was scored as class *e* for the *spoIIIAH* mutant (Fig. 2C). Rather, 36% of *spoIIQ* sporangia and 49% of *spoIIIAH* sporangia were scored as class *b‐c* (Fig. 2C). As a result, the number of class *d* cells (engulfment completed) decreased to 10% for the *spoIIQ* mutant, and to 3% for the *spoIIIAH* mutant (Fig. 2C). We conclude that the *spoIIQ* or *spoIIIAH* mutants are both impaired in forespore engulfment.

### *spoIIQ* and *spoIIIAH* mutants form bulges and vesicles during engulfment

Class *b‐c* sporangia of both mutants (*i.e*., scored at an intermediate stage in engulfment) were often seen in which the two edges of the engulfing membranes were asymmetrically located or had bulges or vesicles protruding from the septal region into the mother cell cytoplasm (Supporting Information Fig. S2, red arrows in panels B and C) and that contained DNA (Supporting Information Fig. S2, yellow arrows in the overlay figures of panels B and C). To better characterize these phenotypes, *b‐c* cells were sub‐divided into two additional classes (Fig. 2C and D). Sub‐class *b1* includes sporangia showing bulges, vesicles or asymmetrically located edges of the engulfing membranes (Fig. 2B and C; yellow and green arrows). Sub‐class class *b2* (Fig. 2C and D) groups sporangia that show septa with a zig‐zag shape where bulge formation seems to occur off the center of the septum (Fig. 2B, blue arrows) and septa curved towards the forespore distal pole rather than into the mother cell cytoplasm (Fig. 2B, white arrows). Class *b1* sporangia were found at a frequency of about 38% for *spoIIQ* and of 31% for *spoIIIAH* mutants (Fig. 2B and D; note that the numbers refer to the percentage of cells in class *b‐c* showing the indicated phenotypes). Class *b2* sporangia were seen at a frequency of 0.5% for the *spoIIQ* mutant but at a frequency of 69% for the *spoIIIAH* mutant (Fig. 2B and D). Thus, a distinctive phenotypic characteristic of the *spoIIIAH* mutant is the presence of septa that curved inward, into the forespore, and the formation of bulges off center (Fig. 2B and D).

Bulges and vesicles are not seen for *spoIIQ* or *spoIIIAH* mutants of *B. subtilis*. They are however formed by mutants in the *spoIID*, *spoIIM* or *spoIIP* genes, coding for the DMP complex (Lopez‐Diaz *et al*., 1986; Smith *et al*., 1993; Margolis *et al*., 1993; Frandsen and Stragier, 1995; Aung *et al*., 2007; Gutierrez *et al*., 2010) (Fig. 2D), or in *spoIIB* mutants, where the DMP proteins fail to localize (Perez *et al*., 2000). Septa that curve towards the forespore pole are also seen in *B. subtilis* cells lacking both septal hydrolases SpoIID and SpoIIP or in cells unable to produce σ^E^ and hence lacking any of the DMP proteins (Illing and Errington, 1991a; Rodrigues *et al*., 2013). Moreover, asymmetrically positioned leading edges of the engulfing membranes has also been noted for a *B. subtilis* mutant with impaired activity of the SpoIID lytic transglycosylase (Gutierrez *et al*., 2010). The bulges/vesicles phenotype of DMP mutants in *B. subtilis* is due to the absence of PG hydrolytic activity within the septum that, together with continued synthesis of PG, causes growth of the bulges and eventually formation of the vesicles (Meyer *et al*., 2010). We speculate that the bulges and vesicles seen for the *spoIIQ* and *spoIIIAH* mutants of *C. difficile* may arise by a similar mechanism, i.e., impaired PG hydrolytic activity within the septum whilst biosynthetic activity is maintained (Meyer *et al*., 2010). We suggest that PG degradation within the septum is impaired in *spoIIQ* and *spoIIIAH* mutants of *C. difficile*.

### The H120S substitution in the LytM domain does not cause formation of bulges or vesicles during engulfment

We considered the possibility that the bulges/vesicles phenotype of the *spoIIQ* and *spoIIIAH* mutants of *C. difficile* was caused, at least in part, by the absence of an activity associated with the SpoIIQ protein, which possesses a LytM domain with all the conserved residues needed for the formation of a functional endopeptidase catalytic site (Fig. 1D). To test for a role of the SpoIIQ LytM domain in *C. difficile*, the conserved His120 residue in motif 1 was changed to a serine, found at the homologous position in the enzymatically inactive *B. subtilis* SpoIIQ orthologue (Fig. 1D; see also the Experimental procedures). The *spoIIQ H120S‐SNAP^Cd^* allele, under the control of its normal promoter, was introduced in a plasmid, which was transferred to the *spoIIQ* mutant strain (Fig. 2A). Asymmetric septation and forespore chromosome segregation proceeded normally in the mutant (Supporting Information Fig. S2, compare A with C and D). The analysis of the *spoIIQ H120S* mutant by SR‐SIM following FM4‐64 staining shows that 13% of the sporangia complete engulfment (sum of classes *d* and *e*) as compared to 14% (classes *d* to *f*) for the *spoIIQ*‐*SNAP* ^Cd^ strain (Fig. 2C). This does not differ much from the percentage of class *d* cells seen for a *spoIIQ* deletion mutant (10%; Fig. 2C). Strikingly, however, no bulges, vesicles or inverted septa were seen for the *spoIIQ H120S‐SNAP^Cd^* strain (Fig. 2C, sub‐classes *b1* and *b2*). In experiments detailed below, we found that SpoIIQ^H120S^‐SNAP^Cd^ accumulates to reduced levels as compared to the WT protein, and localizes less efficiently (section on the localization of SpoIIQ‐SNAP^Cd^). Thus, the cells of the *spoIIQ H120S* mutant able to complete engulfment appear to do so independently of an intact LytM domain, or at least of the presence of His120.

### At least one component of the DMP machine localizes independently of SpoIIQ or SpoIIIAH

As an intact LytM domain in SpoIIQ may not be essential for engulfment, it seemed unlikely that the bulges/vesicles phenotype of the *spoIIQ* deletion mutant was due to the loss of an enzymatic activity. Moreover, the bulges/vesicles phenotype is also seen for the *spoIIIAH* mutant, which is not predicted to exhibit any enzymatic activity. Still, the bulges/vesicles phenotype of *spoIIQ* and *spoIIIAH* mutants, reminiscent of the phenotypes associated with DMP mutants of *B. subtilis* (Lopez‐Diaz *et al*., 1986; Smith *et al*., 1993; Margolis *et al*., 1993; Frandsen and Stragier, 1995; Aung *et al*., 2007; Gutierrez *et al*., 2010), suggests that SpoIIQ and SpoIIIAH are somehow required for a PG hydrolytic activity during engulfment. In *B. subtilis*, the primary landmark protein for localization of the DMP machinery is SpoIIB, produced in pre‐divisional cells (Perez *et al*., 2000; Aung *et al*., 2007). However, SpoIIQ and SpoIIIAH can localize the DMP proteins in the absence of SpoIIB *via* an alternative pathway involving SpoIVFA, a membrane protein required for the activation of σ^K^ (Aung *et al*., 2007). As both SpoIIB and SpoIVFA are absent from the *Clostridia* (Galperin *et al*., 2012), it seemed possible that SpoIIQ and SpoIIIAH could be involved in the correct localization of the DMP proteins in *C. difficile*. As a first test of this idea, we constructed a *spoIID‐SNAP^Cd^* fusion and examined the localization of the protein by SR‐SIM. Control experiments showed that under our experimental conditions no release of the SNAP^Cd^ domain by proteolysis was detected (Supporting Information Fig. S3A). In WT cells, SpoIID‐SNAP^Cd^ localizes along the mother cell membranes, but it is enriched at the septal region (Supporting Information Fig. S4A, red and green arrows). SpoIID‐SNAP^Cd^ was found lining flat septa, and as faint foci at the leading edges of the engulfing membranes (Supporting Information Fig. S4A, yellow arrows). The signal from SpoIID‐SNAP^Cd^ remained following engulfment completion (Supporting Information Fig. S4A). Surprisingly, SpoIID‐SNAP^Cd^ remained at the septum in *spoIIQ* or *spoIIIAH* sporangia and in the latter mutant, enrichment at the leading edges of the engulfing membranes was more apparent (Supporting Information Fig. S4B and C). Thus, at least one of the components of the DMP PG hydrolytic machine is recruited to the septal region independently of SpoIIQ or SpoIIIAH. Possibly then, SpoIIQ and SpoIIIAH are directly or indirectly required for proper activity of the DMP complex or of a yet unknown factor required for engulfment (see also the Discussion).

### SpoIIQ‐ and SpoIIQ^H120S^‐SNAP localize to the septum and forespore membranes

**Figure 3:**
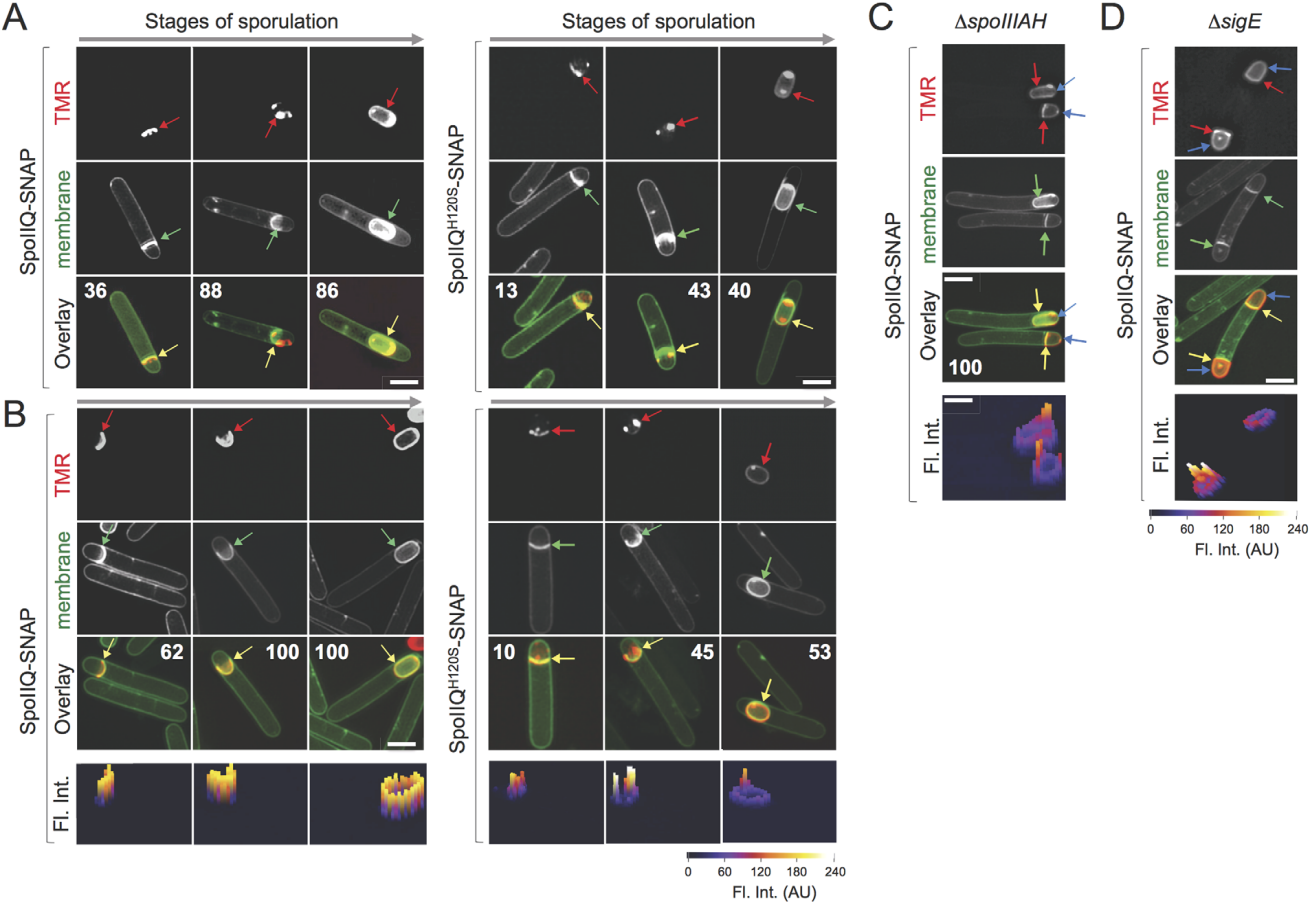
Localization of SpoIIQ‐SNAP^Cd^. The figure illustrates the localization of SpoIIQ‐ and SpoIIQ^H120S^‐SNAP^Cd^ during sporulation, in the presence (A) and in the absence (B) of the WT *spoIIQ* gene, and of SpoIIQ‐SNAP^Cd^ in cells of *spoIIIAH* (C) or *sigE* (D) mutants. The strains expressing the SpoIIQ‐SNAP^Cd^ fusions were grown for 14 hours in SM and samples collected for labelling of the SNAP^Cd^ reporter with TMR‐Star. The labelled cells were viewed by SR‐SIM. The red arrows point to the SNAP^Cd^‐TMR‐Star signal, the green arrows to the septa or engulfing membranes (MTG signal) and the yellow arrows to the merged signal. Note that in panels C and D, the SNAP^Cd^‐TMR‐Star signal is also found ahead of the engulfing membranes (blue arrows). The numbers in the panels are the percentage of cells found with a similar localization pattern at each of the represented stages of sporulation. At least 50 cells were scored for each stage to derive the indicated percentages. Fluorescence intensity profiles (Fl. Int.; scale in arbitrary units) are shown below panels B, C and D. Scale bar, 2 µm.

The analysis of the localization of SpoIID‐SNAP^Cd^ in *spoIIQ* or *spoIIIAH* sporangia suggested that the recruitment and maintenance of SpoIID to the septum and engulfing membranes was essentially independent of SpoIIQ and SpoIIIAH. To gain further insight into the function of SpoIIQ and SpoIIIAH, we first studied the subcellular localization of the SpoIIQ‐SNAP^Cd^ and SpoIIQ^H120S^‐SNAP^Cd^ fusion proteins by SR‐SIM. As shown above, SpoIIQ‐SNAP^Cd^ is largely functional (Table 1). In WT cells, SpoIIQ‐SNAP^Cd^ first localized uniformly along flat septa; then, in cells with curved septa, the fusion proteins form foci at the edges and at the center of the septum (Fig. 3A). In sporangia that had completed the engulfment sequence, SpoIIQ‐SNAP^Cd^ was found around the entire contour of the forespore (Fig. 3A). This pattern did not differ from that seen for SpoIIQ‐SNAP^Cd^ in the absence of *spoIIQ*, except that in the deletion mutant, the frequency of cells decorated by the fusion protein was higher (Fig. 3, compare the percentages shown for cells at different stages in the engulfment sequence in panels A and B, left column). Presumably, SpoIIQ‐SNAP^Cd^ is slightly impaired for assembly at least in the presence of the WT protein. Immunobloting and fluoroimaging analysis of proteins in whole cell extracts before and after labelling with the TMR‐Star substrate shows that more SpoIIQ‐SNAP^Cd^ accumulates in the absence of WT SpoIIQ than in WT cells (Supporting Information Fig. S3B). Thus, it seems possible that, in the presence of WT SpoIIQ, the SpoIIQ‐SNAP^Cd^ protein that fails to localize is degraded.

The localization of the SpoIIQ^H120S^‐SNAP^Cd^ fusion protein followed the same pattern as that of SpoIIQ‐SNAP^Cd^ (along flat septa, foci at the edges of the curved septa, and full encasement of the engulfed forespore) (Fig. 3A and B, right panels). However, the percentage of cells showing localization of SpoIIQ^H120S^‐SNAP^Cd^ in *spoIIQ* mutant or WT cells was lower than for SpoIIQ‐SNAP^Cd^ (Fig. 3; compare the percentages shown in panels A and B, right). Fluorescence intensity profiles suggest that the levels of SpoIIQ^H120S^‐SNAP^Cd^ localized to the septum and engulfing membranes are lower than for the WT fusion protein (Fig. 3B, bottom panels). Moreover, the integrated fluorescence signal localized around engulfed spores was lower for SpoIIQ^H120S^‐SNAP^Cd^ as compared to SpoIIQ‐SNAP^Cd^ (Fig. 3B, bottom panels; 11544 AU for the WT fusion as compared to 5146 AU for SpoIIQ^H120S^‐SNAP^Cd^ for sporangia shown). In agreement with this analysis, immunobloting and fluoroimaging experiments show that SpoIIQ^H120S^‐SNAP^Cd^ accumulated at slightly lower levels than SpoIIQ‐SNAP^Cd^ in either the presence or the absence of WT SpoIIQ (Supporting Information Fig. S3B). The H120S substitution may have rendered the fusion protein slightly unstable *in vivo*. It is also possible that SpoIIQ^H120S^‐SNAP^Cd^ is partially impaired in its ability to correctly localize and that mislocalized protein is degraded (see also below). However, since the bulges and vesicles phenotype is not seen for the *spoIIQ H120S‐SNAP^Cd^* strain, it seems likely that once at its subcellular address, the protein is largely functional during engulfment. While the highest spore titer obtained for the complementation of the *spoIIQ* mutation with the *spoIIQ‐SNAP^Cd^* allele was of 2.9 × 10^5^ ± 4.4 × 10^4^ spores/ml of culture 72 h after inoculation in SM (see above), the highest spore titer observed for the *spoIIQ*/*spoIIQ H120S‐SNAP^Cd^* strain, also at hour 72, was of only 7.83 × 10^3^ ± 2.4 × 10^2^ spores/ml (Table 1). Thus, *spoIIQ H120S‐SNAP^Cd^* does not support sporulation efficiently. Impaired localization and/or instability of the SpoIIQ^H120S^‐SNAP^Cd^ protein may explain its failure to fully restore sporulation to a *spoIIQ* mutant. Because the *spoIIQ H120S‐SNAP^Cd^* strain sometimes completes the engulfment sequence, it appears that SpoIIQ bearing an intact LytM domain is required for a late, post‐engulfment completion, stage of sporulation.

### Co‐dependent localization of SpoIIQ/SpoIIQ^H120S^‐ and SpoIIIAH‐SNAP

We then wanted to determine whether the localization of SpoIIQ‐SNAP^Cd^ was dependent on the presence of SpoIIIAH. For that, the plasmid expressing *spoIIQ‐SNAP^Cd^* was introduced into the *spoIIIAH* mutant, and sporulating cells imaged by SR‐SIM, following MTG staining. In the absence of SpoIIIAH, SpoIIQ‐SNAP^Cd^ was still detected at the septum, but the protein also showed dispersed localization around the forespore, even in cells with flat septa (Fig. 3C, red and blue arrows). Fluorescence intensity profiles confirmed the presence of SpoIIQ‐SNAP^Cd^ ahead of the engulfing membranes (Fig. 3C, bottom). The mislocalization of SpoIIQ‐SNAP^Cd^ was particularly evident in the abortive disporic sporangia formed by *sigE* mutants, in which SpoIIIAH and the DMP components are not produced, and a second polar septum is formed (Fig. 3D). The localization of SpoIIQ in *B. subtilis* is partly dependent on *spoIIIAH* (Rubio and Pogliano, 2004; Blaylock *et al*., 2004; Doan *et al*., 2009; Fredlund *et al*., 2013; Rodrigues *et al*., 2013).

**Figure 4:**
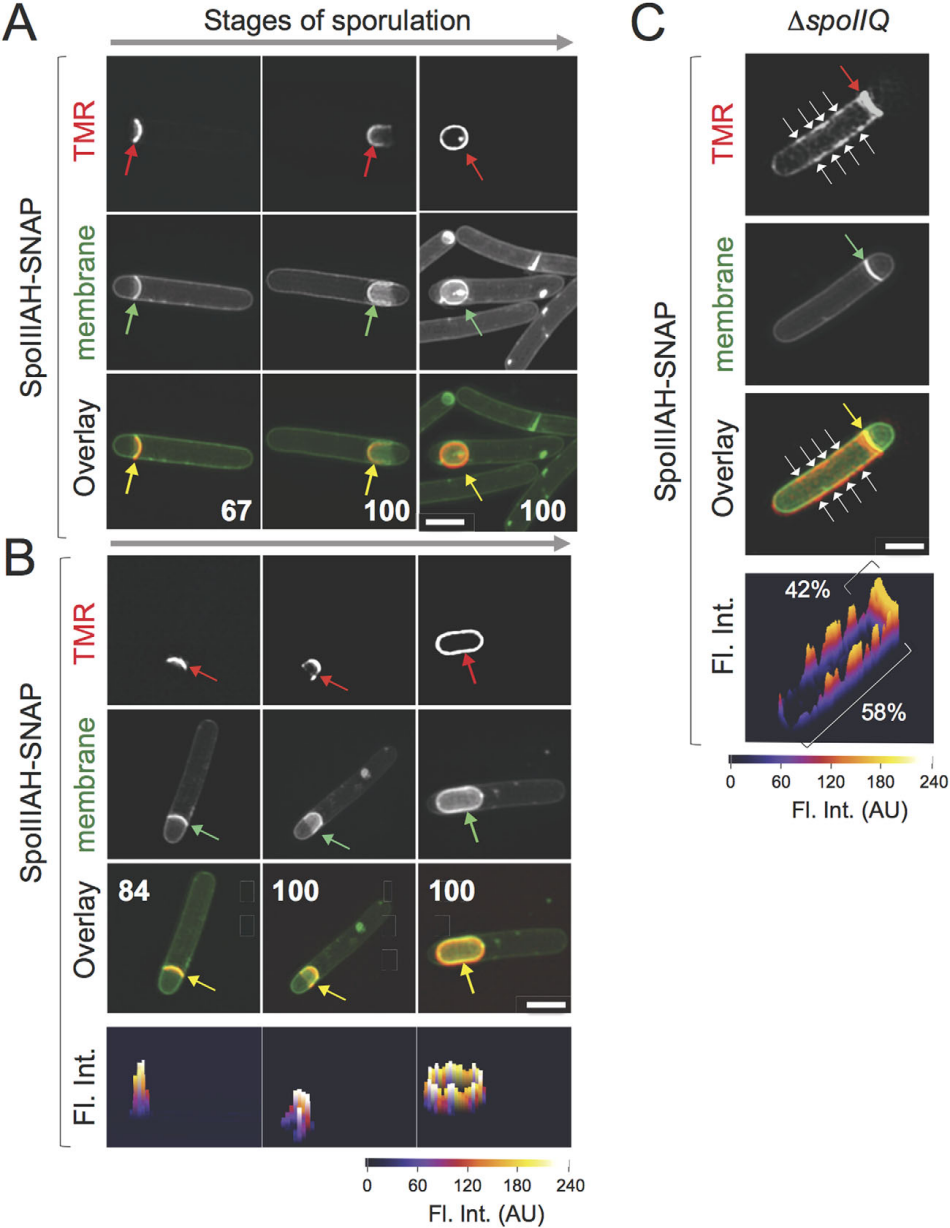
Localization of SpoIIIAH‐SNAP^Cd^. The figure illustrates the localization of SpoIIIAH‐SNAP^Cd^ fusion protein in sporulating cells in the presence (A) and in the absence (B) of the WT *spoIIIAH* allele, and in cells of a *spoIIQ* deletion mutant (C). The strains were grown for 14 hours in SM and samples collected for labelling of the SNAP^Cd^ with TMR‐Star, and the labelled cells were imaged by SR‐SIM. The red arrows in panels point to the SNAP^Cd^‐TMR‐Star signal, the green arrows to the asymmetric septa or engulfing membranes, and the yellow arrows to the merged signals. The red arrow in panel C indicates the TMR‐Star signal enriched at the septum and the white arrows the signal detected throughout the mother cell membrane. The numbers in the panels indicated the percentage of cells found with a similar localization pattern at each of the represented stages of sporulation. At least 50 cells were scored for each stage to derive the indicated percentages. Fluorescence intensity profiles (Fl. Int.; the scale is in arbitrary units) are shown below panels B and D. Scale bar, 2 µm.

Next, we examined the localization of SpoIIIAH‐SNAP^Cd^. The fusion protein decorated a smaller percentage of sporangia in the presence of the WT *spoIIIAH* allele in cells that had just initiated engulfment but not in sporangia at later stages in the sequence (Fig. 4A, compare the percentages in the top and bottom set of panels). As assessed by its ability to restore sporulation to the *spoIIIAH* mutant, SpoIIIAH‐SNAP^Cd^ is largely functional (Table 1). Under our conditions, no release of the SNAP^Cd^ domain from SpoIIIAH‐SNAP^Cd^ was detected (Supporting Information Fig. S3C). Reminiscent of the localization pattern of SpoIIQ‐SNAP^Cd^, the SpoIIIAH‐SNAP^Cd^ fusion protein localized along flat septa and was found around the forespore in sporangia in which engulfment was completed (Fig. 4B). However, at intermediate stages in engulfment, while SpoIIQ‐SNAP^Cd^ formed foci at the leading edges of the engulfment membranes in the presence of the WT *spoIIQ* allele, SpoIIIAH‐SNAP^Cd^ formed a continuous shell or arc lining the curved, migrating membrane, in both the presence or in the absence of the *spoIIIAH* WT allele (Fig. 4B). In agreement, fluorescence intensity profiles show little enrichment of SpoIIIAH‐SNAP^Cd^ at the leading edges of the engulfing membranes (Fig. 4B, bottom panels). Thus, the data suggests that not all SpoIIIAH co‐localizes with SpoIIQ.

Consistent with this inference, while SpoIIIAH‐SNAP^Cd^ mislocalized along the mother cell membrane in the absence of SpoIIQ (Fig. 4C, white arrows), it was still found at the septal plate (yellow arrows). Fluorescence intensity profiles reveal a pattern of strong SpoIIIAH‐SNAP^Cd^ signals along the mother cell membrane, suggesting that some of the delocalized protein remains associated (Fig. 4C, bottom). Integration of the fluorescence signal suggests that about 42% of the fusion protein remains at the septum (Fig. 4C, bottom panel). Therefore, although SpoIIQ and SpoIIIAH are largely co‐dependent for localization, some SpoIIIAH can localize to asymmetric septa and the engulfing membranes independently of SpoIIQ. This contrasts with the situation in *B. subtilis*, where the localization of SpoIIIAH is dependent on SpoIIQ, but SpoIIQ localizes in the absence of SpoIIIAH (Rubio and Pogliano, 2004; Rodrigues *et al*., 2013; Fredlund *et al*., 2013). It suggests that in addition to SpoIIQ, tethering of SpoIIIAH at the septum and engulfing membranes involves additional factors.

### SpoIIQ and SpoIIIAH form a complex in vivo

Both the pattern and the partial co‐dependency for localization of SpoIIQ and SpoIIIAH suggested that the two proteins interact, as is the case for *B. subtilis* (Blaylock *et al*., 2004; Doan *et al*., 2009). To test for formation of a complex between SpoIIQ and SpoIIIAH, we took advantage of the strains producing the SpoIIQ‐SNAP^Cd^ or SpoIIQ^H120S^‐SNAP^Cd^ translational fusions, and the SNAP‐Capture resin in a modified pull‐down assay (see the Experimental procedures section). The SNAP‐Capture resin carries the SNAP substrate benzylguanine covalently cross‐linked to agarose beads, which allows the SNAP tag to be covalently bound to the resin. We prepared whole cell lysates from SM cultures of the strains producing the various fusion proteins in our standard sampling conditions (Figs. 3 and 4). As a control, we also prepared a lysate from a strain expressing the isolated SNAP^Cd^ domain under the control of the mother cell‐specific P_*spoIIIA*_ promoter (Supporting Information Fig. S5, ‘SNAP’). The lysates were incubated with the SNAP capture resin, the beads were washed and the proteins interacting with the immobilized SNAP fusion released by boiling and analysed by immunoblotting with anti‐SNAP or anti‐SpoIIIAH antibodies. Note that in these experiments the immobilized ‘Spo’‐SNAP^Cd^ fusion is not released from the column by boiling and therefore is not resolved by SDS‐PAGE. Using an anti‐SNAP antibody, we verified accumulation of the SNAP domain, of SpoIIQ‐SNAP^Cd^ and of SpoIIQ^H120S^‐SNAP^Cd^ in the whole cell extracts (Supporting Information Fig. S5, bottom panel). However, SpoIIQ^H120S^‐SNAP^Cd^ (but not SpoIIQ‐SNAP^Cd^) remained undetected (Supporting Information Fig. S4). This is in line with the inference that the H120S substitution may impair the *in vivo* stability of the fusion protein. Following incubation of the beads with the lysates, the mixture was washed. Some SNAP^Cd^ or SpoIIQ‐SNAP^Cd^ was detected in the wash fractions for the two assays indicating incomplete capture by the beads (Supporting Information Fig. S5, bottom). After boiling, no SNAP or SpoIIQ‐SNAP^Cd^ was detected by immunoblotting, consistent with covalent binding of the proteins to the SNAP substrate immobilized on the beads.

Importantly, SpoIIIAH, detected with a specific antibody raised against the purified extracytoplasmic domain of the protein (Supporting Information Fig. S6; see also the Experimental Procedures section), was pulled down by SpoIIQ‐SNAP^Cd^ but not by the isolated SNAP^Cd^ domain, indicating binding to the immobilized fusion protein (Supporting Information Fig. S5, top). However, the presence of SpoIIIAH in the wash fraction of the assay suggests that not all of the SpoIIIAH present in the extract binds to the immobilized SpoIIQ‐SNAP^Cd^, or that not all SpoIIIAH molecules bind with equal strength to the fusion protein. SpoIIIAH was not detected in the pull‐down fraction of the SpoIIQ^H120S^‐SNAP^Cd^‐producing strain (Supporting Information Fig. S5, top, last lane), possibly due to the low levels of accumulation of the fusion protein. Also, the levels of SpoIIIAH in the extracts and wash fraction of the strain producing SpoIIQ^H120S^‐SNAP^Cd^ were reduced compared to the strain producing the WT SpoIIQ‐SNAP^Cd^ fusion (Supporting Information Fig. S5, top). It is possible that a less stable complex is formed between SpoIIQ^H120S^‐SNAP^Cd^ and SpoIIIAH, as suggested by our *in vitro* experiments described below, and that unbound SpoIIIAH (and also SpoIIQ^H120S^; see above) is less stable. Our failure to produce an antibody against the purified SpoIIQ protein precluded a pull‐down experiment with immobilized SpoIIIAH‐SNAP^Cd^. In any event, the results show that at least a fraction of the SpoIIIAH molecules forms a complex with SpoIIQ‐SNAP^Cd^ in sporulating cells of *C. difficile*.

### *C. difficile* SpoIIQ is a Zn^2+^‐binding protein

**Figure 5:**
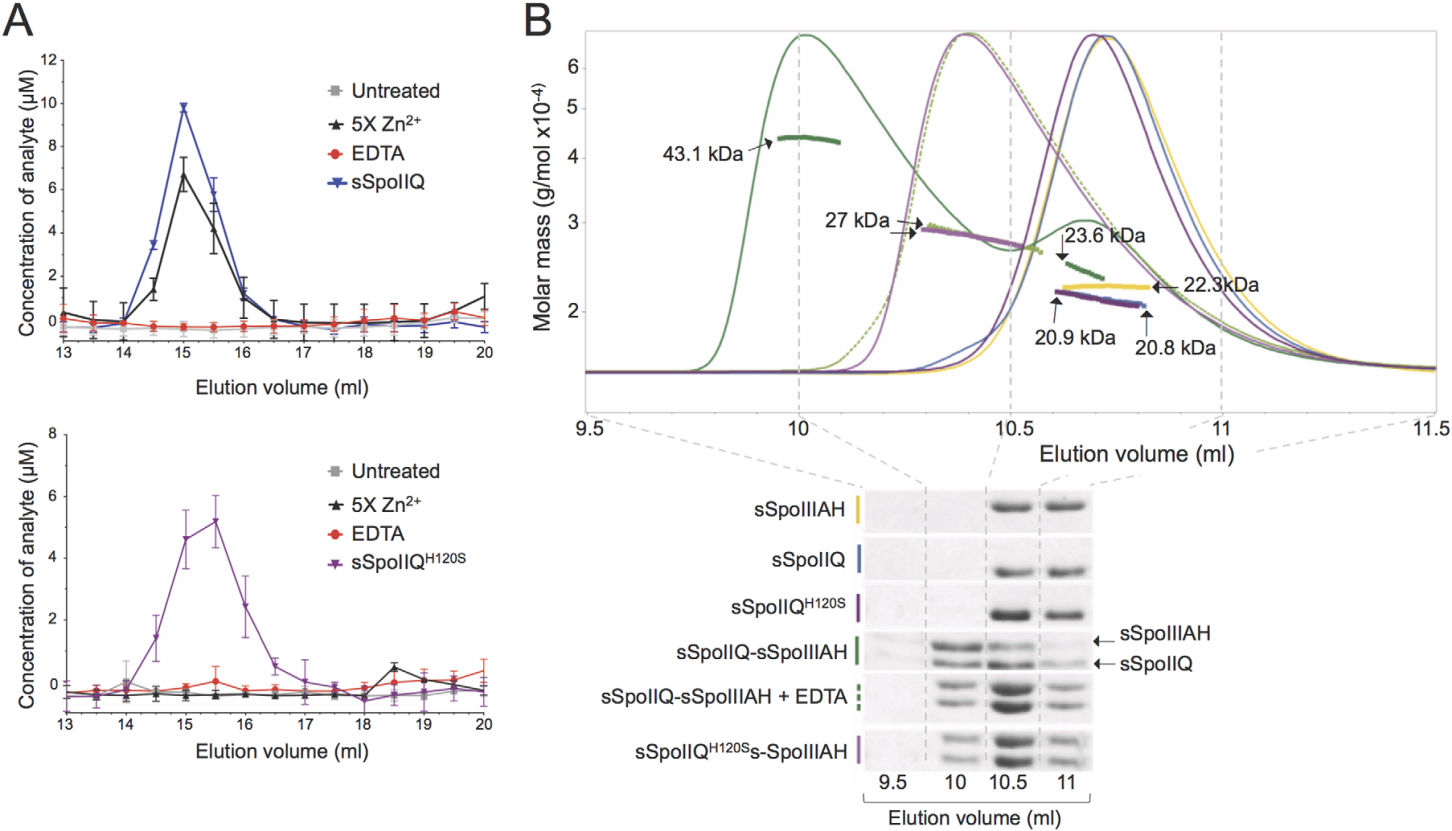
The interaction of SpoIIQ with SpoIIIAH *in vitro*. A. Quantification of Zn^2+^ content in sSpoIIQ (top) and sSpoIIQ^H120S^ (bottom). Purified protein samples were analyzed by size exclusion chromatography and the metal content in each fraction determined using ICP‐MS (see Material and Methods for details), whilst protein concentration in each fraction was determined by absorbance at 280 nm. The content of Zn^2+^ was determined for untreated samples (gray trace in both panels), in the presence of fivefold excess of Zn^2+^ (black traces) or 1mM EDTA (red traces) as purified protein samples eluted from the size exclusion column. In order to estimate the metal occupancy in each sample, protein quantification in each sample is also shown (sSpoIIQ, blue trace, top; sSpoIIQ^H120S^, purple trace, bottom). In the presence of an excess Zn^2+^, about 80% sSpoIIQ (top panel, blue vs back traces) coordinates the metal, whilst no metal binding is detected for the sSpoIIQ^H120S^ mutant (bottom panel, purple vs black traces). B. sSpoIIQ interacts with sSpoIIIAH in a zinc‐associated mechanism. Purified sSpoIIQ (blue trace), sSpoIIQ^H120S^ (purple trace) and sSpoIIIAH (yellow trace) were analyzed by SEC‐MALLS. Traces correspond to light scattering measurements throughout protein elution. All proteins elute as single monomeric species, as indicated by the calculated masses (blue, purple and yellow dotted lines) and confirmed by SDS‐PAGE analysis (bottom, rows 1–3 respectively). Dotted lines correspond to the weight average molecular mass calculated based on refractive index and light scattering measurements. Complex sSpoIIQ‐sSpoIIIAH (1:1 molar ratio, green trace) elutes with a calculated mass of 43.1 kDa (green dotted line). Surprisingly, lack of coordination of Zn^2+^ seems to result in a less stable complex, as the sSpoIIQ^H120S^‐sSpoIIIAH (light purple trace) and the WT complex incubated with 1mM EDTA (dashed light green trace) elute with a calculated mass of ∼27 kDa. Fractions (0.5ml) corresponding to elution volumes between 9.5 and 11ml were analyzed by SDS‐PAGE for all chromatography experiments (bottom, dashed gray lines indicate start and end of collection for each fraction).

Unlike its *B. subtilis* counterpart, the SpoIIQ protein of *C. difficile* has all the residues required to form a LytM domain able to bind Zn^2+^ ([Crawshaw *et al*., 2014]; Fig. 1D and Supporting Information Fig. S7). Moreover, the observed phenotypes of the *spoIIQ H120S* mutant indicate that an intact LytM domain is important for the function of SpoIIQ, at least following engulfment completion (above). As a first step to investigate the behavior of SpoIIQ and SpoIIIAH *in vitro*, we wanted to establish whether SpoIIQ in *C. difficile* has the ability to coordinate Zn^2+^. We analyzed the Zn^2+^ content of pure protein samples by inductively coupled plasma mass spectrometry (ICP‐MS). The soluble domains of the WT and H120S variants of SpoIIQ (sSpoIIQ and sSpoIIQ^H120S^, residues 31–222; i.e. where the N‐terminal transmembrane domain is replaced by a TEV – tobacco etch virus protease‐cleavable His_6_ tag; Supporting Information Fig. S6), were overproduced in *E. coli* and purified by Ni^2+^‐affinity chromatography, followed by tag removal (see Material and Methods for details). Samples of purified protein were then analyzed by size exclusion chromatography (SEC) immediately after affinity chromatography (Fig. 5A, untreated, grey trace), as well as after incubation with a fivefold Zn^2+^ excess (Fig. 5A, 5x Zn^2+^, black trace) or in the presence of 1mM EDTA (Fig. 5A, EDTA, red trace). Fractions from the SEC column were collected and the metal content quantified. In order to establish the relative occupancy with Zn^2+^, the protein concentration for each fraction was also determined (Fig. 5A, top, sSpoIIQ, blue trace and bottom panel, sSpoIIQ^H120S^, purple trace). Addition of an excess of Zn^2+^ shows that sSpoIIQ can bind to the metal to approximately 80% occupancy, assuming that each sSpoIIQ molecule binds one Zn^2+^ ion (Fig. 5A, compare the blue and black traces). As expected, the H120S substitution abolishes binding to the metal, even in the presence of excess Zn^2+^ (Fig. 5A, bottom panel, compare purple and black traces). The absence of detectable metal in the untreated samples is likely due to the affinity purification steps, where imidazole would have chelated the metal, as well as partial metal chelation by the Superdex resin. These results show that *C. difficile* SpoIIQ is a Zn^2+^ binding protein and that His120 is required for metal coordination.

### The interaction between SpoIIQ and SpoIIIAH *in vitro* is influenced by Zn^2+^

To determine whether *C. difficile* SpoIIQ and SpoIIIAH interact directly, the purified proteins were analysed by size exclusion chromatography multi‐angle laser light scattering (SEC‐MALLS), alone or in combination. The soluble domain of SpoIIIAH (sSpoIIIAH, residues 29–229) was overproduced and purified as described above for sSpoIIQ and sSpoIIQ^H120S^ (Supporting Information Fig. S6; for details see the Experimental procedures section). We first analyzed the behaviour of the individual proteins, which eluted as single peaks with calculated molecular masses of 22.3 kDa for sSpoIIIAH, 20.9 kDa for sSpoIIQ, and 20.8 kDa for sSpoIIQ^H120S^ (Fig. 5B, top, yellow, blue and purple dotted lines respectively, which correspond to the weight average molecular mass, calculated from refractive index and light scattering measurements). As the predicted MW of these proteins is 23.0 kDa for sSpoIIIAH and 21.4 kDa for both forms of sSpoIIQ, the calculated masses indicate that all proteins are monomeric in solution. Analysis by SDS‐PAGE of the elution fractions (Fig. 5B, bottom panel, first three rows) confirms the presence of a single species in solution.

Next, purified sSpoIIQ and sSpoIIIAH were incubated for one hour in a 1:1 molar ratio, in the presence of a fivefold excess of Zn^2+^ to allow for maximum metal binding, and analyzed by SEC‐MALLS. This analysis revealed a new species eluting at 10.0 ml (Fig. 5B, green trace), with a calculated mass of 43.1 kDa (Fig. 5B, green dotted line) and containing both proteins, as verified by SDS‐PAGE (Fig. 5B, bottom panel, row 4). These results confirm that SpoIIQ and SpoIIIAH interact *via* their soluble domains, as observed for the *B. subtilis* orthologues (Meisner *et al*., 2012; Levdikov *et al*., 2012). The calculated molecular mass of the complex suggests the presence of a heterodimer of sSpoIIQ and sSpoIIIAH. An additional peak was observed with a calculated mass of 23.6 kDa (Fig. 5B, second peak between 10.5 and 11 ml, green trace and dotted line). Analysis by SDS‐PAGE indicates that this peak corresponds mostly to sSpoIIQ, with only traces of sSpoIIIAH detectable in the gel (Fig. 5B, bottom, row 4). This could be a result of an excess of sSpoIIQ in the original incubation due to differences in protein concentration calculations and/or some partial dissociation of the complex.

Surprisingly, incubation of sSpoIIQ^H120S^ with sSpoIIIAH at a 1:1 molar ratio, yielded a different elution profile (Fig. 5B, top, light purple trace), although a complex was still formed, as detected by SDS‐PAGE (Fig. 5B, bottom, last lane). This complex elutes with a calculated MW of 27.3 kDa (Fig. 5B, top, light purple dotted line), a value between that expected for a heterodimeric species and for monomeric sSpoIIQ^H120S^ or sSpoIIIAH, indicating the presence of a partially dissociating complex. Consistent with the localization of SpoIIQ^H120S^‐SNAP^Cd^, which depends on SpoIIIAH, this result confirms that sSpoIIQ^H120S^ still interacts with sSpoIIIAH but that the complex formed seems to be less stable. Together, these observations imply that His120 and/or coordination of Zn^2+^ impacts on the interaction of SpoIIQ with SpoIIIAH.

To test whether the observed effect is due to the ability of SpoIIQ to bind Zn^2+^ and not to an indirect effect of the H120S substitution, the SEC‐MALLS analysis of purified WT sSpoIIQ and sSpoIIIAH was repeated after incubation of the proteins in the presence of 1 mM EDTA. As shown by ICP‐MS experiments, purified sSpoIIQ contains no detectable zinc and addition of EDTA ensures no metal ions would be present in solution. Importantly, the presence of EDTA does not alter the elution profile of sSpoIIQ alone (Supporting Information Fig. S6C). In the absence of metals, the sSpoIIQ‐sSpoIIIAH complex now exhibits an elution profile identical to that of the sSpoIIQ^H120S^‐sSpoIIIAH sample, with a calculated mass of 27.6 kDa (Fig. 5B, top, light green dashed trace and line; bottom row 5). This confirms that the presence of Zn^2+^ alters the characteristics of the complex formed between sSpoIIQ and sSpoIIIAH, and that when sSpoIIQ is unable to bind the metal, the interaction of the two proteins is affected.

### SpoIIQ and SpoIIIAH interact throughout engulfment

**Figure 6:**
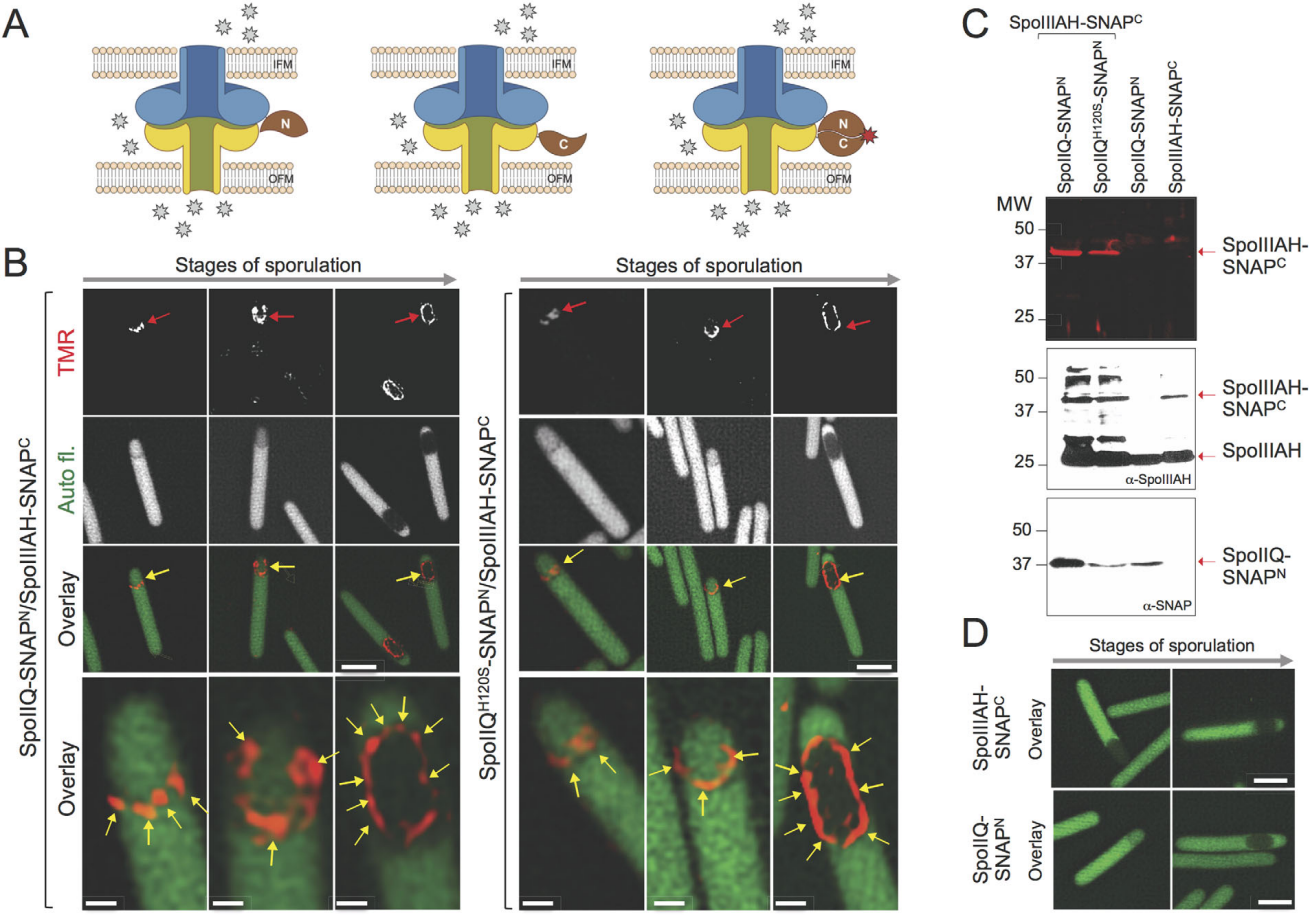
Split‐SNAP localization. A. The top diagrams depict the predicted topology of SpoIIQ‐SNAP^Cd‐N^ (Q) and SpoIIIAH‐SNAP^Cd‐C^ fusions (AH) in the forespore membranes and their interaction based on the *B. subtilis* model. Assuming that in *C. difficile* the proteins will form a complex with a similar topology, a fluorescent signal (red star when bound to the reconstructed SNAP domain) will only be observed if the proteins interact. Note that the cells express the fusions (SpoIIQ‐SNAP^Cd‐N^ or SpoIIQ^H120S^‐SNAP^Cd‐N^ under the control of P_*spoIIQ*_ promoter and SpoIIIAH‐SNAP^Cd‐C^ under P_*spoIIIA*_ control) from the same plasmid in an otherwise WT background. Thus, not all the subunits of the putative SpoIIQ‐SNAP^Cd‐N^ or SpoIIIAH‐SNAP^Cd‐C^ oligomers may be labelled. For simplicity, only one subunit is shown fused to the reporter. B. The figures show the localization of the fluorescence signal from the reconstituted SNAP^Cd^ domain (red arrows), in cells expressing the indicated combinations of SNAP^Cd‐N^/SNAP^Cd‐C^ fusions. The cells were grown for 14 hours in SM prior to sampling, labelling with TMR‐Star and SR‐SIM imaging. The bottom series of panels are a magnification of the cells marked with an arrow in the ‘overlay’ panels; the yellow arrows point to foci of SNAP‐TMR‐Star. Scale bar, 2 µm. C. Split‐SNAP accumulation. Whole cell extracts were prepared from sporulating cells (same conditions as in A), producing SpoIIQ‐SNAP^Cd‐N^ or SpoIIQ^H120S^‐SNAP^Cd‐N^ and SpoIIIAH‐SNAP^Cd‐C^ (first two lanes), or the SpoIIQ‐SNAP^Cd‐N^ and SpoIIIAH‐SNAP^Cd‐C^ proteins alone in an otherwise WT background. Proteins in the extracts were resolved by SDS‐PAGE, subjected to fluoroimaging (top panel) and immunobloting with anti‐SpoIIIAH (middle panel) or anti‐SNAP (bottom) antibodies. The red arrows show the position of SpoIIIAH‐SNAP^Cd‐C^, SpoIIIAH or SpoIIQ‐SNAP^Cd‐N^. Note that only SpoIIIAH‐SNAP^Cd‐C^ retains the covalently bound TMR‐Star substrate following SDS‐PAGE. Molecular weight markers (in kDa) are shown to the right side of the panel. D: fluorescence microscopy of cells producing SpoIIIAH‐SNAP^Cd‐C^ or SpoIIQ‐SNAP^Cd‐N^ alone and labelled with SNAP‐TMR‐Star. The labelled cells were examined by fluorescence microscopy in the green (auto‐fluorescence) and red channels. Note that the forespore is identified by its reduced auto‐fluorescence signal. Scale bar, 2 µm.

In an attempt to localize the interaction between SpoIIQ and SpoIIIAH in sporulating cells, we made use of a Split‐SNAP reporter (Mie *et al*., 2012). The crystal structure of the SNAP‐tag shows that the protein consists of two domains (Daniels *et al*., 2000). Earlier studies have shown that the 128‐residue SNAP‐tag can be split into an N‐terminal fragment (residues 1–91) and a C‐terminal fragment (residues 92–182) carrying the reactive Cys145 residue, that do not re‐associate efficiently unless fused to two interacting proteins (Fang *et al*., 2005; Mie *et al*., 2012). We fused the C‐terminus of SpoIIQ to the N‐terminal moiety of SNAP^Cd^ (or SNAP^Cd‐N^) and the C‐terminus of SpoIIIAH to the C‐terminal moiety of the reporter (SNAP^Cd‐C^) (Fig. 6A). Under these conditions and based on the *B. subtilis* model, the interaction of SpoIIQ and SpoIIIAH would allow association of the SNAP^Cd^ N‐ and C‐terminal domains in the intermembrane space, formation of the substrate‐binding site, which includes Cys145 in the C‐terminal moiety, and labelling of the reconstituted reporter (Fig. 6A).

Co‐production of SpoIIQ‐SNAP^Cd‐N^ with SpoIIIAH‐SNAP^Cd‐C^ resulted in a fluorescence signal from the reconstituted SNAP^Cd^ domain along flat septa (Fig. 6B, red arrows). Fluorescence from the reconstituted SNAP^Cd^ was also seen in sporangia with curved septa as a punctate pattern along the engulfing membranes (Fig. 6B, yellow arrows), reminiscent of the pattern of SpoIIQ‐ and SpoIIIAH‐SNAP^Cd^ localization (Figs. 3 and 4; above). These foci, which are more evident on the higher magnification images shown as the bottom row of figure 6B, presumably represent SpoIIQ‐SpoIIIAH complexes. In the higher magnification images, nearly circular signals are seen, whose size (≤120 nm across) is too large to represent SpoIIQ‐SpoIIIAH oligomers, assuming that the structure proposed for the *B. subtilis* channel is conserved (Meisner and Moran, 2011; Levdikov *et al*., 2012; Meisner *et al*., 2012,) (Fig. 6A). The origin of these structures is presently unclear. Decoration of the entire contour of the forespore following engulfment completion was also detected and the punctate pattern persisted (Fig. 6B, last column of the set of panels on the left). We also examined the interaction between SpoIIQ^H120S^‐SNAP^Cd‐N^ and SpoIIIAH‐SNAP^Cd‐C^. A punctate pattern of the fluorescence signal from the reconstituted SNAP^Cd^ was also found at septa, engulfing membranes and around the engulfed forespore but only in about 43% of the sporangia, as compared to 71% for the strain producing SpoIIQ‐SNAP^Cd‐N^ (Fig. 6B, set of panels on the right).

In whole cell extracts prepared from the samples used for fluorescence microscopy, SpoIIQ‐SNAP^Cd‐N^ or SpoIIQ^H120S^‐SNAP^Cd‐N^ accumulate as a species of approximately 37 kDa, as detected with an anti‐SNAP monoclonal antibody, consistent with the predicted size of the fusions (Fig. 6C). SpoIIIAH was detected with an anti‐SpoIIIAH antibody as a species of about 25 kDa, and SpoIIIAH‐SNAP^Cd^ as a species of about 40 kDa, consistent with their predicted size (Fig. 6C). SpoIIIAH‐SNAP^Cd‐C^ was not detected with the anti‐SNAP monoclonal antibody, suggesting that this antibody only recognizes the N‐terminal domain of the SNAP tag. The difference in mobility between the SpoIIQ‐SNAP^Cd‐N^ (or SpoIIQ^H120S^‐SNAP^Cd‐N^) and SpoIIIAH‐SNAP^Cd‐C^ fusions is also consistent with the slower migration of purified SpoIIIAH relative to SpoIIQ in SDS‐PAGE (Supporting Information Fig. S6B). Neither SpoIIQ‐SNAP^Cd‐N^ nor SpoIIIAH‐SNAP^Cd‐C^ was labelled by the TMR‐Star substrate when independently produced, as detected by fluoroimaging (Fig. 6C, last two lanes) or fluorescence microscopy (Fig. 6D). In contrast, SpoIIIAH‐SNAP^Cd‐C^, which carries Cys145, was labelled when the fusion was co‐produced with either SpoIIQ‐SNAP^Cd^ or SpoIIQ^H120S^‐SNAP^Cd^ (Fig. 6C, first two lanes).

Thus, SpoIIQ‐SNAP^Cd^ and SpoIIQ^H120S^‐SNAP^Cd^ interact with SpoIIIAH‐SNAP^Cd^ in the septum, appear to form discrete foci during engulfment, and remain associated upon engulfment completion. The observed interaction of SpoIIQ^H120S^‐SNAP^Cd‐N^ with SpoIIIAH‐SNAP^Cd‐C^ in the forespore membranes is consistent with formation of a complex between sSpoIIQ^H120S^ and sSpoIIIAH, albeit altered, *in vitro* (see above).

### *spoIIQ* and *spoIIIAH* are required for late forespore‐ and mother cell‐specific gene expression

**Figure 7:**
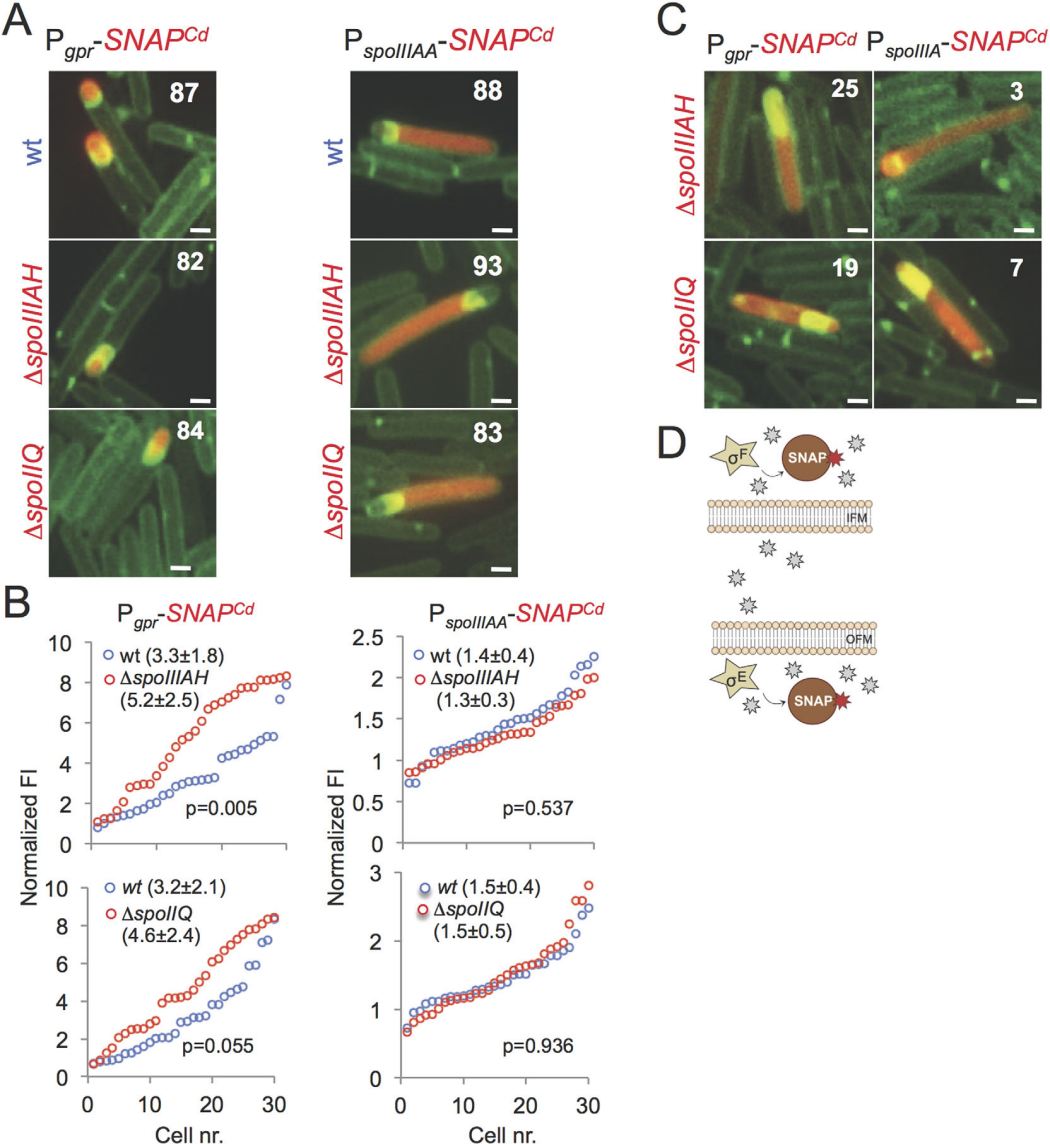
Early cell type‐specific gene expression in *spoIIQ* and *spoIIIAH* mutants. A. Cells expressing transcriptional P_*gpr*_‐*SNAP^Cd^* (as a reporter for σ^F^ activity) or P_*spoIIIA*_‐*SNAP^Cd^* fusions (as a reporter for σ^E^ activity) in the WT or the congenic *spoIIQ* or *spoIIIAH* mutants were collected from SM cultures 14 hours after inoculation, stained with the SNAP substrate TMR‐Star, and examined by phase contrast and fluorescence microscopy. The numbers represent the percentage of cells with the indicated pattern, relative to the total number of sporulating cells. A minimum of 50 sporangia was scored for each panel. Scale bar, 2 µm. B. Quantification of the fluorescence signal for the indicated SNAP^Cd^ fusions in the WT (in cells that had not completed the engulfment sequence), and in the *spoIIQ* or *spoIIIAH* mutants, as indicated. C. The panel illustrates *spoIIQ* or *spoIIIAH* sporangia in which loss of compartmentalized activity of the P_*gpr*_‐ and P_*spoIIIA*_‐*SNAP^CD^* fusions is seen; the numbers represent the percentage of sporangia with the indicated pattern relative to the number of cells in A showing expression of either fusion. D. Early cell‐type specific gene expression is unaffected in *spoIIQ* and *spoIIIAH* mutants. In the absence of SpoIIQ or of SpoIIIAH (neither protein is represented for simplicity), σ^F^ and σ^E^ show essentially normal activity (the active sigma factors are shown inside a star), as seen by labelling of the SNAP reporter with TMR‐Star (stars; red when protein bound).

A critical function of the mother cell to forespore channel formed by SpoIIQ and SpoIIIAH in *B. subtilis* is to allow continued transcription in the forespore when, following engulfment completion, it becomes isolated from the external environment (Camp and Losick, 2009). As at least some cells of the *spoIIQ H120S* mutant complete engulfment without the accumulation of abnormal forms such as bulges, vesicles and inverted septa (Fig. 2C), but the mutant is still impaired in sporulation (Table 1), we reasoned that SpoIIQ and possibly SpoIIIAH could also have a post‐engulfment function in sporulation. To test this possibility, we examined whether the *spoIIQ* and *spoIIIAH* mutations had an effect on early and late gene expression in *C. difficile*. We monitored the activity of σ^F^ and σ^E^ using transcriptional fusions of the *gpr* and *spoIIIA* promoters to the *SNAP^Cd^* tag respectively (Saujet *et al*., 2013; Pereira *et al*., 2013). Expression of P_*gpr*_‐*SNAP^Cd^* was detected in 87% of the sporulating cells of the WT, in 82% of *spoIIIAH* sporangia, and in 84% of the *spoIIQ* sporangia (Fig. 7A). Expression of P_*spoIIIA*_‐*SNAP^Cd^* was detected in 88% of the sporulating cells in the WT, in 93% of the cells in the *spoIIIAH* mutant and 83% of the *spoIIQ* sporangia (Fig. 7A). A quantitative analysis revealed an increase in the intensity of the fluorescence signal for the P_*gpr*_‐*SNAP^Cd^* fusion in individual cells of the *spoIIQ* or *spoIIIAH* mutants, prior to engulfment completion (Fig. 7B, left column of plots). This increase may result from augmented activity of σ^F^ in sporangia impaired in the engulfment sequence and/or from a reduction in the activity of σ^G^ prior to engulfment completion ([Pereira *et al*., 2013b]; see also below). In contrast, no effect of the mutations was detected for the P_*spoIIIA*_‐*SNAP^Cd^* fusion (right column of plots). We conclude that deletion of *spoIIIAH* or *spoIIQ* does not curtail the activity of the early forespore‐ and mother cell‐specific σ factors during engulfment. Nevertheless, we found whole cell expression of the P_*gpr*_‐*SNAP^Cd^* fusion in 25% of the *spoIIIAH* sporangia and in 19% of the *spoIIQ* cells, and of the P_*spoIIIA*_‐*SNAP^Cd^* fusion in 3% and 7% of the *spoIIIAH* and *spoIIQ* sporangia respectively (Fig. 7C). This loss of compartmentalization of the activities of σ^F^ and σ^E^ is most likely due to instability and eventual collapse of the forespore membranes in either mutant (as seen in Fig. 2B and Supporting Information Fig. S2). Instability of the forespore membranes in *spoIIQ* and *spoIIIAH* sporangia has also been reported for *B. subtilis* (Doan *et al*., 2009).

**Figure 8:**
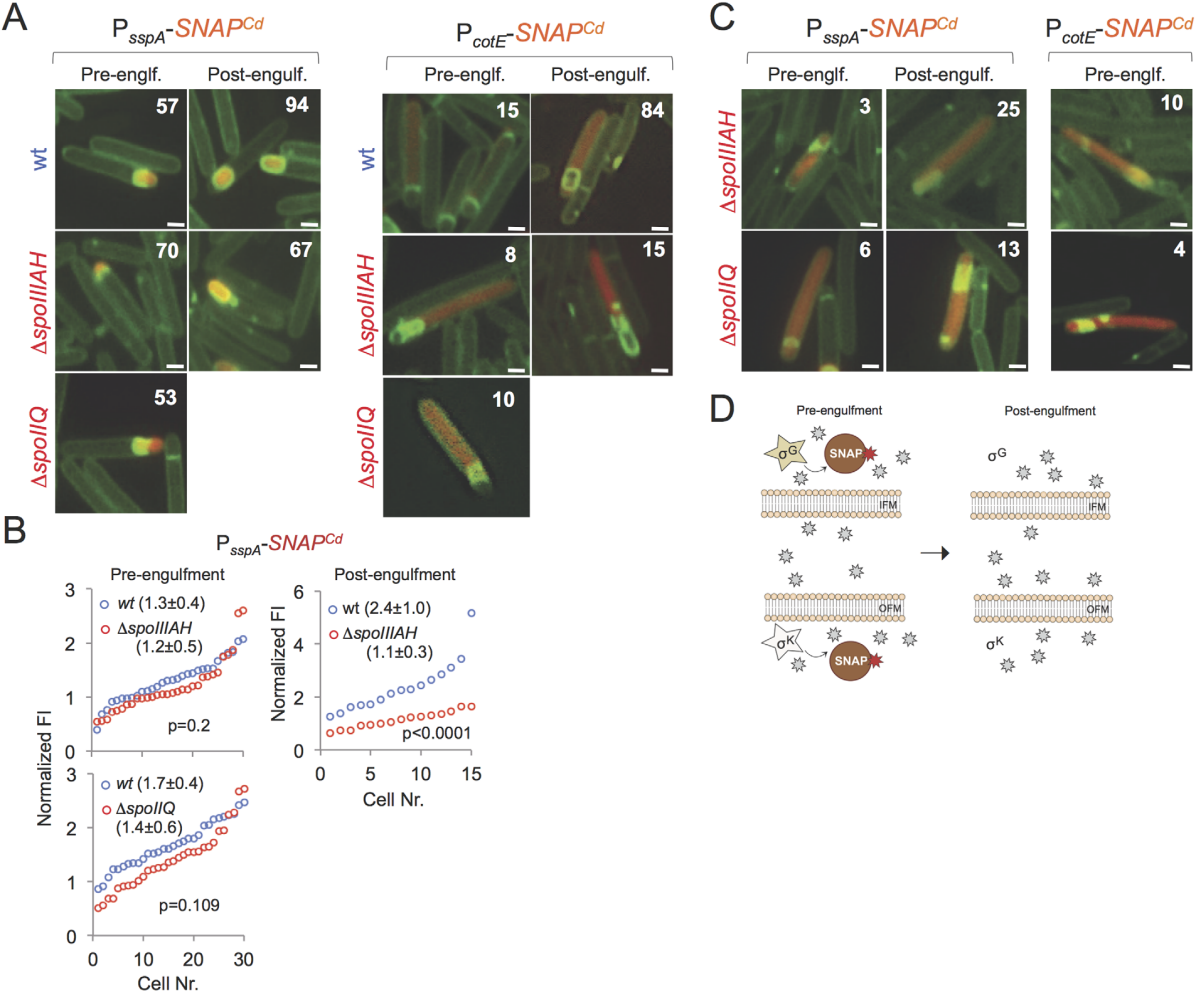
Late cell type‐specific gene expression in *spoIIQ* and *spoIIIAH* mutants. A. Cells expressing transcriptional P_*sspA*_‐*SNAP^Cd^* (as a reporter for σ^G^ activity) or P_*cotE*_‐*SNAP^Cd^* fusions (as a reporter for σ^K^ activity) in an otherwise WT background or in congenic *spoIIQ* or *spoIIIAH* mutants were collected from SM cultures 14 hours after inoculation, stained with the SNAP substrate TMR‐Star, and examined by phase contrast and fluorescence microscopy. The numbers represent the percentage of cells, relative to the total number of sporulating cells, exhibiting expression of P_*sspA*_‐*SNAP^Cd^* (as a reporter for the activity of σ^G^) or P_*cotE*_‐*SNAP^Cd^* (as a reporter for the activity of σ^K^). A minimum of 50 sporangia was scored for each panel. Scale bar, 1 µm. B. Quantification of the fluorescence signal for the P_*sspA*_‐*SNAP^Cd^* fusion in the WT (blue) and in the *spoIIQ* or *spoIIIAH* mutants (red, as indicated). The two left panels show the fluorescence signal in cells prior to engulfment completion. The right panel shows the fluorescence signal for cells of the *spoIIIAH* mutant following engulfment completion. C. Lack of *spoIIQ* or *spoIIIAH* leads to loss of compartmentalized activity of the P_*sspA*_‐ or P_*cotE*_‐*SNAP^CD^* fusions. The numbers indicate the percentage of sporangia in A showing fluorescence from P_*sspA*_‐ or P_*cotE*_‐*SNAP^CD^* in both the forespore and the mother cell. D. Late cell‐type specific gene expression is reduced in *spoIIQ* and *spoIIIAH* mutants. In the absence of SpoIIQ or of SpoIIIAH (not represented), the post‐engulfment activity of both σ^G^ (forespore) and σ^K^ (mother cell), as assessed by production and labelling of the SNAP reporter, is severely curtailed (the active σ factors are shown inside a star). In addition, the fraction of cells that display σ^K^ activity prior to engulfment completion is reduced (light grey star).

To monitor the activity of σ^G^ and σ^K^ we used the previously characterized P_*sspA*_‐SNAP^Cd^ and P_*cotE*_‐SNAP^Cd^ transcriptional fusions respectively (Pereira *et al*., 2013). While σ^G^ and σ^K^ show some activity during engulfment, their main period of activity begins with engulfment completion (Pereira *et al*., 2013). Expression of the σ^G^‐dependent P_*sspA*_‐*SNAP^Cd^* fusion was detected in 57% of the sporangia in the WT, in 70% of the *spoIIIAH* cells and in 53% of the *spoIIQ* cells prior to engulfment completion (Fig. 8A). Moreover, neither mutation significantly affected the distribution of the fluorescence signal measured in individual cells during engulfment; the average signal intensity was of 1.3 ± 0.4 for the WT and of 1.2 ± 0.5 for the *spoIIIAH* mutant (*P* = 0.2) and of 1.4 ± 0.6 for *spoIIQ* cells in comparison with 1.7 ± 0.4 for the WT, in a parallel experiment (*P* = 0.109) (Fig. 8B). The slight reduction in the activity of σ^G^ in *spoIIQ* or *spoIIIAH* sporangia prior to engulfment completion, as assessed using the P_*sspA*_‐*SNAP^Cd^* reporter, may nevertheless contribute to the increased activity of σ^F^, as suggested above. However, in cells of the *spoIIIAH* mutant in which engulfment was completed, the distribution of the fluorescence signal in individual cells was strongly reduced (Fig. 8B; average intensity, in arbitrary units, of 2.4 ± 1.0 for the WT, and of 1.1 ± 0.3 for the mutant; *P* < 0.0001). We did not quantify the intensity of the signal for the fraction of *spoIIQ* cells expressing the P_*sspA*_‐*SNAP^Cd^* fusion, as they were rare in the population. In addition, for the *spoIIIAH* mutant, 3% of the cells scored during engulfment and 25% of the cells scored following engulfment completion P_*sspA*_‐*SNAP^Cd^* displayed a whole cell expression pattern (Fig. 8C). This whole cell pattern of fluorescence was also seen in 6% of the *spoIIQ* sporangia during engulfment and in 13% of the cells that had completed engulfment (Fig. 8C). Whole cell fluorescence is presumably due to collapse of the forespore membranes and loss of compartmentalization (Fig. 8C) (Doan *et al*., 2009).

As shown in figure 8A, the number of pre‐engulfment cells expressing the σ^K^ reporter fusion P_*cotE*_‐*SNAP^Cd^* fusion only represented 8% of the sporulating cells in the *spoIIIAH* mutant and 10% of the *spoIIQ* cells (as compared with 15% for the WT), and we did not quantify the intensity of the fluorescence signal in those cells (Fig. 8A, right set of panels). A whole cell pattern of P_*cotE*_‐*SNAP^Cd^* expression was detected for 10% of the *spoIIIAH* cells and 4% of the *spoIIQ* cells prior to engulfment completion, most likely due to instability of the forespore membranes as suggested above for a similar pattern observed for the P_*sspA*_‐*SNAP^Cd^* fusion (Fig. 8C). The *spoIIQ* and *spoIIIAH* mutations thus appear to reduce slightly the fraction of pre‐engulfment cells in which σ^K^ is active. Strikingly, however, 84% of the WT cells that had concluded the engulfment sequence expressed the P_*cotE*_‐*SNAP^Cd^* reporter, whereas in the *spoIIIAH* mutant only 15% of the cells expressed the fusion (Fig. 8A). Scoring was not possible for cells of the *spoIIQ* mutant in which very few cells showing complete engulfment were seen (see also above).

We conclude that the *spoIIQ* and *spoIIIAH* mutations severely compromise the activity of the forespore‐specific σ^G^ factor following engulfment completion (Fig. 8D). Furthermore, while both the *spoIIQ* and *spoIIIAH* deletion mutations reduced the activity of σ^K^ during engulfment, at least the *spoIIIAH* deletion mutation strongly curtailed the activity of σ^K^ following engulfment completion (Fig. 8D).

## Discussion

We show that the *spoIIQ* and *spoIIIAH* genes of *C. difficile* are required for sporulation because they have a role during engulfment of the forespore by the mother cell and are additionally needed for late stages of morphogenesis, following engulfment completion. Our observations bear important implications for our understanding of the engulfment process, and the control of gene expression during endospore development.

### Engulfment

In *B. subtilis*, forespore engulfment is mainly controlled by the DMP machine, while PG synthesis and the SpoIIQ‐SpoIIIAH interaction play non‐essential, redundant roles (Crawshaw *et al*., 2014). The contribution of the SpoIIQ‐SpoIIIAH interaction for engulfment and its more stringent requirement under certain nutritional conditions suggests that redundancy confers environmental robustness to the process (Broder and Pogliano, 2006). It has also been suggested that the SpoIIQ‐SpoIIIAH interaction may represent an original core mechanism for engulfment, present before the emergence of the more complex DMP‐based process (Ojkic *et al*., 2014). Our results are in line with this inference, as we found that *spoIIQ* or *spoIIIAH* mutants of *C. difficile* are impaired in engulfment. However, *spoIIQ* and *spoIIIAH* mutants show phenotypes reminiscent of those associated with DMP mutants of *B. subtilis* (Lopez‐Diaz *et al*., 1986; Margolis *et al*., 1993; Smith *et al*., 1993; Frandsen and Stragier, 1995; Aung *et al*., 2007). While the function of the DMP machine in *C. difficile* remains to be characterized, it seems possible that both the DMP and the SpoIIQ‐SpoIIIAH modules are essential for engulfment in *C. difficile* and that these two engulfment modules have become functionally separated in *B. subtilis* and presumably in related organisms. In both *spoIIQ* and *spoIIIAH* mutants of *C. difficile*, bulges and vesicles are formed at the asymmetric septum, reminiscent of similar structures formed in *B. subtilis* by *spoIID* and *spoIIP* mutants which are blocked in the initial stage of engulfment, septal thinning (Lopez‐Diaz *et al*., 1986; Illing and Errington, 1991a; Frandsen and Stragier, 1995; Abanes‐De Mello *et al*., 2002). Bulges and vesicles are sites of active cell wall synthesis and their formation is prevented by cell wall‐active antibiotics as well as by disruption of the gene coding for the SpoVD transpeptidase (which itself localizes to the bulges and vesicles) (Meyer *et al*., 2010). We presume that the bulges and vesicles arise in the *C. difficile* mutants by impaired cell wall degradation activity and continued synthesis of new cell wall as described for *B. subtilis* (Meyer *et al*., 2010). Since the DMP proteins are conserved in *C. difficile*, one possibility is that deletion of *spoIIQ* or *spoIIIAH* may somehow impair the activity of the DMP machine. This control may not operate at the level of the localization of the DMP complex as at least a SpoIID‐SNAP^Cd^ translational fusion localized to the asymmetric septum in *spoIIQ* or *spoIIIAH* sporangia (Supporting Information Fig. S4). However, it remains to be tested whether the localization of the SpoIIM and SpoIIP protein is affected in *spoIIQ* or *spoIIIAH* mutants. In *B. subtilis*, the septal localization of the DMP proteins is mainly controlled by SpoIIB, with SpoIIQ and SpoIIIAH proteins making an indirect contribution. A SpoIIB orthologue is absent from most *Clostridia* including *C. difficile* (Galperin *et al*., 2012) and how SpoIID‐SNAP^Cd^ localizes to the septum in the absence of SpoIIQ or SpoIIIAH is not known. Consistent with a role in engulfment, SpoIIQ‐SNAP^Cd^ and SpoIIIAH‐SNAP^Cd^ fusions localize to the septum and the engulfing membranes. Moreover, SpoIIQ‐SNAP^Cd^ formed a complex with SpoIIIAH *in vivo*, and Split‐SNAP^Cd^ fusions suggest a direct interaction between the two proteins during engulfment. However, while the localization of SpoIIQ‐SNAP^Cd^ is dependent on SpoIIIAH, the localization of SpoIIIAH‐SNAP^Cd^ to the septum is only partially dependent on SpoIIQ. In *B. subtilis*, it is the localization of SpoIIQ that is partially independent of SpoIIIAH, and requires the DMP machine, or a product generated by its activity (Rubio and Pogliano, 2004; Blaylock *et al*., 2004; Doan *et al*., 2005; Fredlund *et al*., 2013; Rodrigues *et al*., 2013). Possibly, the localization of SpoIIIAH in *C. difficile* requires, in addition to SpoIIQ, the DMP proteins or a product of their activity. The bulges/vesicles phenotype is more pronounced in *spoIIIAH* cells, in which septa that curve towards the spore pole are a distinctive phenotype. That this particular phenotype was also seen for *B. subtilis spoIID*/*spoIIP* double mutants or in *sigE* sporangia, unable to produce the DMP proteins (Illing and Errington, 1991a; Rodrigues *et al*., 2013) suggests that, in *C. difficile*, SpoIIIAH may have a more prominent role, partly independent of SpoIIQ, in controlling the activity of the DMP machine.

In *B. subtilis*, engulfment is much faster in the absence (1‐2 min; dependent on SpoIIQ/SpoIIIAH) than in the presence of the cell wall (about 45 min; dependent on DMP) (Ojkic *et al*., 2014). However, in the absence of the cell wall, about 40% of the engulfing membranes retract (Ojkic *et al*., 2014). Since in *C. difficile* the SpoIIQ‐SpoIIIAH complex has a more central role in engulfment, it will be interesting to investigate the mechanics and kinetics of engulfment in this organism.

### Role of the LytM domain of SpoIIQ in spore morphogenesis

SpoIIQ has an intact LytM endopeptidase domain suggesting that the protein could directly participate in PG hydrolysis during engulfment. However, in *B. subtilis* and in most other *Bacillus* species and closely related organisms (Crawshaw *et al*., 2014) (Fig. 1D), the LytM domain of SpoIIQ lacks a conserved histidine residue required for coordination of Zn^2+^ as part of the catalytic center (Supporting Information Fig. S7A). At least in *B. subtilis* SpoIIQ, where a serine replaces the conserved histidine residue, the protein no longer coordinates the metal and seems to be catalytically inactive (Supporting Information Fig. S7B, compare top and bottom; see also the Supporting Results and Discussion). The observation that in *C. difficile* and in several other *Clostridia*, the histidine residue is present raised the possibility that, in these organisms, an enzymatically active SpoIIQ protein directly contributed to PG hydrolysis during engulfment. Importantly, we show that WT SpoIIQ, but not a form in which the conserved histidine was replaced by a serine, coordinates Zn^2+^ and therefore could exhibit enzymatic activity. However, the *spoIIQ H120S* allele did not completely impair progress through the engulfment sequence, and in particular, did not cause a bulge/vesicle phenotype. Thus, intact LytM motifs able to bind Zn^2+^ may not be essential during engulfment in *C. difficile*.

In *B. subtilis*, the LytM domain is involved in the interaction with SpoIIIAH and also with a yet unidentified protein that contributes to the septal localization of SpoIIQ, at least in the absence of SpoIIIAH (Rodrigues *et al*., 2013). Using SEC‐MALLS, we show that *C. difficile* C‐terminal domains sSpoIIQ and sSpoIIIAH form a heterodimeric complex in solution. Importantly, we show that Zn^2+^ binding by SpoIIQ plays a currently uncharacterized role in the interaction with SpoIIIAH. Firstly, the complex formed by sSpoIIQ^H120S^ or WT sSpoIIQ in the presence of EDTA is less stable, as seen by SEC‐MALLS. Secondly, an interaction between SpoIIQ^H120S^ and SpoIIIAH is still detected *in vivo* using Split‐SNAP fusions, although in a reduced number of cells.

In *B. subtilis*, SpoIIQ and SpoIIIAH interact *via* their degenerate LytM and YscJ domains respectively (Meisner and Moran, 2011; Levdikov *et al*., 2012; Meisner *et al*., 2012), with the noncatalytic serine downstream from the last β‐sheet of the complex interface (Supporting Information Fig. S7C, see also the Supporting Results and Discussion). Since the H120S substitution affects the stability of the SpoIIQ and SpoIIIAH complex, it seems possible that the LytM catalytic region is involved in the interaction with SpoIIIAH also in *C. difficile*. This hypothesis is further supported by the fact that secondary structure predictions for *C. difficile* SpoIIQ indicate that the protein is less structured than its *B. subtilis* orthologue, particularly in the region around the complex interface (Supporting Information Fig. S7D and Supporting Results and Discussion). Importantly, an α‐helix in *B. subtilis* SpoIIQ shown to stabilize the complex interface is absent in the *C. difficil*e protein (Supporting Information Fig. S7C and D; Supporting Results and Discussion). Moreover, based on channel topology models proposed (Meisner and Moran, 2011; Levdikov *et al*., 2012; Meisner *et al*., 2012), it is possible that these structural elements upstream of the LytM domain might also be involved in the interactions between heterodimers required to form a ring (for details, see Supporting Information S1 text). It is therefore possible that the less structured *C. difficile* SpoIIQ requires coordination by the Zn^2+^ ion to provide a stable platform for interaction with SpoIIIAH and the formation of a stable ring (Supporting Information Fig. S7 and Supporting Results and Discussion).

Work in *B. subtilis* has shown the involvement of SpoIIQ and the *spoIIIA‐*encoded proteins in formation of a specialized secretion machine, through which the mother cell nurtures the developing spore, providing small molecules required for continued transcription in the forespore following engulfment completion (Meisner *et al*., 2008; Camp and Losick, 2009; Doan *et al*., 2009). The second, later function of the SpoIIQ‐SpoIIIAH complex of *C. difficile* in spore morphogenesis may be similar and a functional LytM domain and/or a stable complex could be crucial for this later activity. Further work, including structural determination of WT and SpoIIQ^H120S^ complexes with SpoIIIAH, would help elucidate the exact functional and/or structural role of both Zn^2+^ coordination and an intact LytM domain.

### Expanding the mother cell‐to‐forespore channel model

The specialized secretion system formed by the *spoIIIA‐*encoded proteins and SpoIIQ maintains the stability of the forespore membranes and the potential for macromolecular synthesis in the forespore during late stages of morphogenesis (Serrano *et al*., 2004; Meisner *et al*., 2008; Doan *et al*., 2009; Camp and Losick, 2009). In *B. subtilis*, the transcriptional activity in the forespore following engulfment completion is severely curtailed in *spoIIQ* or *spoIIIAH* mutants, and in these mutants the forespore membranes collapse and compartmentalized gene expression is lost (Serrano *et al*., 2004; Camp and Losick, 2009; Doan *et al*., 2009). Two lines of evidence suggest that the SpoIIQ and SpoIIIAH proteins of *C. difficile* may also support a channel‐like function. Firstly, among the cells of *spoIIQ* or *spoIIIAH* mutants that reach late stages in engulfment or complete the sequence, many show disorganized forespore membranes (Fig. 2 and Supporting Information Fig. S2), and studies with cell type‐specific transcriptional fusions to the SNAP fluorescent reporter show that in those cells compartmentalized gene expression is lost (Figs. 7 and 8). Therefore, like in *B. subtilis*, SpoIIQ and SpoIIIAH are both required for stability of the forespore at late stages of morphogenesis. Secondly, we have shown that mutations in either *spoIIQ* or *spoIIIAH* impair the activity of the late forespore‐specific regulatory protein σ^G^, consistent with the model that the SpoIIQ and SpoIIIAH proteins are required for continued transcription in the forespore. Importantly, the early activities of σ^F^, σ^E^ or σ^G^ were not affected. In *B. subtilis* the onset of σ^G^ activity coincides with engulfment completion. In *C. difficile* however, although the main period of σ^G^ activity follows engulfment completion, σ^G^‐dependent transcription is also detected in the forespore before engulfment is completed (Pereira *et al*., 2013; Saujet *et al*., 2013).

The channel model predicts transport of yet unidentified small molecules into the forespore, required for continued macromolecular synthesis and transcriptional activity in this cell following engulfment completion. In *B. subtilis*, assembly of the channel is also required for late, σ^K^‐dependent transcriptional activity in the mother cell for at least two reasons. Not only is the assembly of the channel required for proper localization of the pro‐σ^K^ processing machinery to the forespore outer membrane, but also the activation of the pro‐σ^K^ processing machinery requires the production, under the control of σ^G^, of a protein secreted to the intermembrane space. Thus, in *B. subtilis*, the activity of σ^K^ is tightly coupled to engulfment completion. In contrast, the activity of σ^K^ in *C. difficile* is detected during engulfment, although it increases following engulfment completion (Fimlaid *et al*., 2013; Saujet *et al*., 2013; Pereira *et al*., 2013). However, the σ^K^ protein of *C. difficile* lacks a pro‐sequence, and the activity of σ^K^ in either pre‐ or post‐engulfment sporangia is independent of σ^G^ (Saujet *et al*., 2013; Pereira *et al*., 2013; Fimlaid *et al*., 2013). Consistent with the absence of a requirement for post‐translational processing by sporulation‐specific proteins, σ^K^ is active upon induction in vegetative cells of *C. difficile* (Pishdadian *et al*., 2015). An important finding of our investigation is that the activity of σ^K^ in *C. difficile*, in spite of its independence on σ^G^, is also impaired before or after engulfment completion in *spoIIQ* or *spoIIIAH* sporangia. In the framework of the channel model, one possible explanation for this observation is that, in the absence of channel activity, a metabolite accumulates in the mother cell that is inhibitory for σ^K^ or alternatively, that the forespore produces a compound required in the mother cell. If so, why σ^E^, which is highly similar to σ^K^, is immune to a putative inhibitory signal or otherwise does not require a forespore‐produced compound, is unclear.

We cannot presently rule out a direct role of SpoIIQ and SpoIIIAH in the regulation of σ^K^ activity. Importantly, it was previously noticed that disruption of the *sigF* gene had an impact on the expression of σ^K^ dependent genes, whereas mutation of σ^G^ essentially had no effect (Saujet *et al*., 2013). Presumably, the impact of the *sigF* mutation on the expression of σ^K^ genes may be accounted for by the lack of *spoIIQ* expression in the mutant, as we now show. How SpoIIQ and SpoIIIAH influence the activity of σ^K^ in *C. difficile* appears to imply a novel regulatory mechanism and is an important goal for future research. In any case, the observation that proper gene expression in the mother cell is also impaired in the absence of SpoIIQ or SpoIIIAH, expands on the channel model, suggests an unexpected degree of metabolic cooperation between the two cells throughout development and may lead to experimental strategies to identify the compound or compounds that are transferred through the channel. As highlighted by this and other recent studies (reviewed in (Paredes‐Sabja *et al*., 2014; Al‐Hinai *et al*., 2015; Fimlaid and Shen, 2015), work in the ancient *Clostridia* group of organisms will continue to provide novel insights into the evolution and mechanism governing endosporulation by the Firmicutes. On the other hand, the SpoIIQ‐SpoIIIAH complex of *C. difficile* represents a promising target for strategies aiming at interfering with sporulation and therefore with the transmission and environmental persistence of this pathogen.

## Experimental procedures

### Growth conditions and general methods

Bacterial strains and their relevant properties are listed in Supporting Information Table S1. The *Escherichia coli* strain DH5α (Bethesda Research laboratories) was used for molecular cloning and strain HB101 (RP4) was used as the donor in conjugation experiments (Hussain *et al*., 2005). Luria‐Bertani medium was routinely used for growth and maintenance of *E. coli*. When indicated, ampicillin (100 µg/ml) or chloramphenicol (15 µg/ml) was added to the culture medium. The *C. difficile* strains used in this study are congenic derivatives of the wild‐type strain 630Δ*erm* (Hussain *et al*., 2005) and were routinely grown anaerobically (5% H_2_, 15% CO_2_, 80% N_2_) at 37°C in Brain Heart Infusion (BHI) medium (Difco) or SM medium (for 1l: 90 g Bacto‐tryptone, 5 g Bacto‐peptone, 1 g (NH_4_)_2_SO_4_ and 1.5 g Tris base) (Wilson *et al*., 1982). When necessary, cefoxitin (25 µg/ml), thiamphenicol (15 µg/ml), or erythromycin (5 µg/ml) was added to *C. difficile* cultures. The efficiency of sporulation was determined as described before (Pereira *et al*., 2013b); see also the Supporting Experimental Procedures.

### Mutants and SNAP^Cd^ fusions

The construction of *spoIIQ, spoIIQH120S and spoIIIAH* mutants and the construction of transcriptional and translational SNAP^Cd^ fusions are described in detail in the Supporting Experimental Procedures.

### Split‐SNAP^Cd^ fusions

The *SNAP^Cd^* was divided between amino acid residues 91 and 92 (Daniels and Tainer, 2000; Mie *et al*., 2012). The N‐ and C‐terminal fragments are referred to as *SNAP^Cd‐N^* and *SNAP^Cd‐C^* respectively. To construct a *spoIIQ*‐*SNAP^Cd‐N^* translational fusion, the *spoIIQ* gene fused to *SNAP^Cd‐N^* was PCR‐amplified from pMS480 using primers spoIIQ‐40D and nSNAPR (all primers used in this study are listed in Supporting Information Table S2) producing a 1288 bp product. A fusion of *spoIIIAH* to *SNAP^Cd‐C^* was constructed by overlap extension. The *spoIIIAH* and c*SNAP^Cd^* were amplified separately from pMS481, using primer pairs spoIIIAAD/P4, and SpoIIIAH‐c*SNAP^Cd^* D/c*SNAP^Cd^* R. The resulting 1222 bp *spoIIIAH* fragment (which includes its native promoter) was mixed with the 275 bp c*SNAP^Cd^* fragment and the mixture amplified using primers spoIIIAAD and c*SNAP^Cd^* R, a step that yielded a fragment of 1497 bp. The *spoIIQ*‐n*SNAP^Cd^* fragment (which includes the native *spoIIQ* promoter) was digested with EcoRI and NotI, mixed with the *spoIIIAH*‐c*SNAP^Cd^* fragment digested with EcoRI and XhoI, and both fragments introduced via a triple ligation into pMTL84121 (Heap *et al*., 2009), digested with NotI and XhoI, creating pMS490. We used pMS490, containing the *spoIIQ* gene (see above) and *spoIIQ*‐specific primers to convert the histidine codon 120 to a serine codon, producing pMS498. All plasmids were introduced into *E. coli* HB101 (RP4) and then transferred to *C. difficile* 630Δ*erm* and derivatives by conjugation (Heap *et al*., 2007) (Supporting Information Table S1).

### SNAP imaging

Whole cell extracts were obtained by withdrawing 5 ml samples from *C. difficile* cultures 14 hours after inoculation in SM medium. The extracts were prepared immediately following labellling with 250 nM of the TMR‐Star substrate (New England Biolabs), for 30 min in the dark. Following labelling, the cells were collected by centrifugation (4000 × *g*, for 5 min at 4°C), the cell sediment was washed with phosphate‐buffered saline (PBS) and re‐suspended in 1 ml French press buffer (10 mM Tris pH 8.0, 10 mM MgCl2, 0.5 mM EDTA, 0.2 mM NaCl, 10% Glycerol, 1 mM PMSF). The cells were lysed using a French pressure cell (18000 lb/in^2^). Proteins in the extracts were resolved on 15% SDS‐PAGE gels. The gels were first scanned in a Fuji TLA‐5100 fluorimager, and then subject to immunoblot analysis as described previously (Pereira *et al*., 2013). The anti‐SNAP antibody (New England Biolabs) was used at a 1:1000 dilution, and a rabbit secondary antibody conjugated to horseradish peroxidase (Sigma) was used at dilution 1:10 000. The immunoblots were developed with enhanced chemiluminescence reagents (Amersham Pharmacia Biotech).

### SNAP‐capture pull‐down experiments

Whole cell extracts were obtained by withdrawing 5 ml samples from *C. difficile* cultures 14 hours after inoculation in SM medium. The cell pellets were re‐suspended in 1ml portions of buffer A [100 mM NaCl, 10 mM Tris‐HCl (pH 8.0), 10% glycerol] and lysed in a French pressure cell (18 000 lb/in^2^). The lysate was cleared by centrifugation. One milliliter of cleared lysate was bound to 80 µl of a 50% slurry of SNAP‐Capture pull down resin (NEB) at room temperature for 30 min. The resin was washed three times in buffer B (same as A but with 200 mM NaCl) and re‐suspended in a final volume of 40 µl. The samples were subjected to SDS‐PAGE and immunoblotting.

### Microscopy

Samples of 1 ml were withdrawn from SM cultures 14 h after inoculation, and the cells collected by centrifugation (4000 × *g* for 5 min). The cells were washed with 1ml of PBS, and re‐suspended in 0.1 ml of PBS supplemented with the lipophilic styryl membrane dye *N*‐(3‐triethylammoniumprpyl)−4‐(*p*‐diethylaminophenyl‐hexatrienyl) pyridinium dibromide (FM4‐64; 10 µg/ml) (Vida and Emr, 1995; Pogliano *et al*., 1999) and the DNA stains DAPI (4′,6‐diamidino‐2‐phenylindole; 50 µg/ml) (from Molecular Probes, Invitrogen) or Hoechst 33342 (2′‐[4‐ethoxyphenyl]‐5‐[4‐methyl‐1‐piperazinyl]‐2,5′‐bi‐1H‐benzimidazole trihydrochloride trihydrate) (from Pierce). For SNAP labelling experiments, 200 μl of cells in culture samples were labelled with TMR‐Star (as above), collected by centrifugation (4000 × *g*, 3 min, at room temperature), washed four times with 1 ml of PBS, and finally re‐suspended in 1ml of PBS containing the membrane dye Mitotracker Green (0.5 µg/ml) (Molecular Probes, Invitrogen). For the quantification of gene expression at the single cell level, we used conventional fluorescence microscopy as detailed in the Supporting Experimental Procedures. For the characterization of mutant phenotypes, the cells were imaged using Super‐resolution Structured Illumination Microscopy (SR‐SIM) performed in an Elyra PS.1 Microscope (Zeiss), using a Plan‐Apochromat 63x/1.4 oil DIC M27 objective and a Pco. edge 5.5 camera. Images were acquired using 405 nm (50 mW), 488 nm (100 mW) or 561 nm (100 mW) laser lines, at 5–20% of total potency. The grid periods used were 23 µm, 28 µm or 34 µm for acquisitions with the 405 nm, 488 nm or 561 nm lasers respectively. For each SIM acquisition the corresponding grating was shifted five times and rotated five times, giving a total of 25 acquired frames. Final SIM images were reconstructed employing the ZEN software (black edition, 2012, version 8,1,0,484; Zeiss), using synthetic, channel‐specific Optical Transfer Functions (OTFs). Fluorescence intensity profiles were generated from the microscopy images (red channel, corresponding to TMR‐Star labelling of the various SNAP fusion proteins) using the 3D Surface plotter function of *ImageJ* (http://imagej.nih.gov/ij/).

### SEC‐MALLS

The overproduction and purification of soluble (denoted by the prefix *s*) versions of WT SpoIIQ, its variant SpoIIQ^H120S^ and SpoIIIAH are described in the Supporting Experimental Procedures. Protein concentration was determined by A280 and adjusted to a total concentration of 2.5 mg/ml. Complex samples were formed by pre‐incubation at 1:1 ratio for 1 h at 4°C. Fivefold excess of ZnCl_2_ or 1 mM EDTA was added to individual or complex samples as necessary. Individual and complex samples were then injected on a Wyatt SEC WTC‐050S5 column primed with 50 mM MES pH 6.0, 250 mM NaCl buffer and analyzed using a Wyatt DAWN HELEOS 8 light scattering detector and Optilab T‐rEX refractive index monitor (Wyatt). 0.5 ml fractions were collected and peak fractions were resolved in 15% SDS‐PAGE gels. Weight average molecular mass was calculated based on refractive index and light scattering measurements using Astra 6 software (Wyatt).

### Metal content analysis

Protein samples in 50 mM MES pH 6.0, 250 mM NaCl were diluted to a concentration of 10 μM (untreated samples). When appropriate, 50 μM (5X) ZnCl_2_ or 1mM EDTA was added to the samples before size exclusion chromatography. Each sample was then loaded individually to a Superdex 200 GL 10/300 Increase column (GE Healthcare). To avoid contamination with metal and/or EDTA, the samples were analyzed in the order: EDTA, untreated, 5X Zn^2+^ and the column was primed with 50 mM MES pH 6.0, 250 mM NaCl or 50 mM MES pH 6.0, 250 mM NaCl, 1 mM EDTA as appropriate. 0.5 ml fractions were collected during elution. ICP‐MS samples (3 ml) were prepared by adding 300 μl sample of each fraction to 2.7 ml of 2.5% HNO_3_ containing 20 ppb Ag as an internal standard. Metal standards run before and after analysis of the fractions contained Mg, Mn, Fe, Cu, Ni, Co, Zn at 0, 1, 5, 10, 25, 50, 75 and 100 ppb. The protein concentration of each fraction was determined by absorbance at A280. Three technical replicates were analyzed by SEC followed by ICP‐MS.

## Note added in proof:

While this manuscript was under revision, Fimlaid and co‐authors published an analysis of the role of spoIIQ and spoIIIAH in *C. difficile* (Fimlaid KA, Jensen O, Donnelly ML, Siegrist MS, Shen A (2015) Regulation of Clostridium difficile Spore Formation by the SpoIIQ and SpoIIIA Proteins. PLoS Genet 11(10): e1005562. doi:10.1371/journal.pgen.1005562).

While the main conclusions in the two studies are in general agreement, this study further expands our insights into the function of spoIIQ and spoIIIAH.

## Supporting information

Supporting Information
