# Supplementary material for: The SpoIIQ‐SpoIIIAH complex of C lostridium difficile controls forespore engulfment and late stages of gene expression and spore morphogenesis
Source: Mol Microbiol. 2016 Feb 12;100(1):204–28. doi: 10.1111/mmi.13311 (PMC4982068; doi:10.1111/mmi.13311)
Supplement: Supplementary file 1 — Supporting Information [file MMI-100-204-s001.pdf]

Supporting Information

The SpoIIQ-SpoIIIAH complex of *Clostridium difficile* controls forespore engulfment and late stages of gene expression and spore morphogenesis

Mónica Serrano[1], Adam D. Crawshaw[2#], Marcin Dembek[2,3#], João M. Monteiro[4], Fátima C. Pereira[1§], Mariana Gomes de Pinho[4], Neil F. Fairweather[3], Paula S. Salgado[2]*, and Adriano O. Henriques[1]*

[1]Microbial Development and [4]Bacterial Cell Biology Laboratory, Instituto de Tecnologia Química e Biológica, Universidade Nova de Lisboa, Avenida da República, Estação Agronómica Nacional, Avenida da República, 2780-157 Oeiras, Portugal; [2]Institute for Cell and Molecular Biosciences, Faculty of Medical Sciences, Newcastle University, Newcastle upon Tyne, UK; [3]MRC Centre for Molecular Bacteriology and Infection, Department of Life Sciences, Imperial College London, London, UK

Supporting Experimental Procedures

Sporulation efficiency. In order to compare the sporulation efficiency of the mutants against the WT, a sporulation efficiency assay was carried out with cultures growing in SM medium (Pereira *et al.*, 2013b). To this end, strains were first inoculated into BHIS, grown for 8 hours and diluted to an OD_{580} of 0.01 into SM and incubated for at least 72 hours. For total CFU counts, at each time point (18, 24 and 72 hours after inoculation into SM), serial 10-fold dilutions were prepared and spotted (20 µl drops, in triplicate) onto pre-reduced BHI supplemented with 0.1% taurocholate (Sigma-Aldrich), to

promote efficient spore germination (Wilson *et al.*, 1982). For spore counts, 500 µl samples were taken out of the cabinet and heated at 70°C for 30 min prior to diluting. The data is represented as means ± SD from three technical replicates. The percentage of sporulation was determined as the ratio between the number of spores/ml and the total number of bacteria/ml times 100. The limit of detection of the assay was 50 CFU/ml. Phase contrast microscopic observation of the samples allowed to differentiate between sporulation and germination defects.

Mutant construction. A *spoIIIAH* in-frame deletion mutant was generated using allelic exchange in *C. difficile* 630Δ*erm*Δ*pyrE* as described by Ng *et al.* (Ng *et al.*, 2013). Briefly, the mutant allele was generated by cloning PCR-amplified homology regions upstream and downstream of the desired junction point within the *spoIIIAH* coding region into pMTL-YN3 (Ng *et al.*, 2013). The upstream fragment (597 bp) was generated with primers spoIIIAH72D and spoIIIAH-669R and the downstream fragment (563 bp) was generated with primers spoIIIAH-1225D and spoIIIAH-1788R; the two fragments were joined by overlapping PCR, the resulting fragment cleaved with AscI and SbfI and cloned between the same sites of pMTL-YN3. The resulting plasmid, pMS501, was introduced into *E. coli* HB101 (RP4) and then transferred to 630Δ*erm*Δ*pyrE* by conjugation (Heap *et al.*, 2007) (S1 Table). Following two passages on BHIS agar supplemented with 5 µg/ml uracil, 15 µg/ml thiamphenicol and 250 µg/ml cycloserine, colonies that were noticeably larger (indicative of plasmid integration) were screened by colony PCR to identify single-crossover mutants using primers flanking the upstream and downstream homology regions in conjunction with a plasmid-specific primer (P3 with P2 and P4 with P1) to amplify across the integration junction. Pure,

single crossover mutants were streaked onto *C. difficile* minimal medium (CDMM) supplemented with 5 µg/ml uracil and 2 mg/ml 5-fluoroorotic acid (FOA) to identity clones resulting from plasmid excision. The isolated FOA-resistant colonies were screened by PCR. Double-crossover mutants, in which the mutant allele was successfully integrated yielded products smaller than those seen in WT revertants. In order to restore the *pyrE*+ phenotype, plasmid pMTL-YN1 carrying the WT *pyrE* allele was conjugated into the isolated double-crossover mutants. The resulting colonies were restreaked onto non-supplemented CDMM agar to select for uracil prototrophy indicating successful allele exchange. Successful restoration of the WT *pyrE* allele was confirmed by colony PCR using primers flanking the *pyrE* locus (Fig. S1, P5 and P6) followed by Sanger sequencing of the amplified product.

***SNAP*^{Cd} transcriptional fusions.** Fusions of the *gpr*, *spoIIIA*, *sspA* and *cotE* promoters to the *SNAP*^{Cd} reporter have been described before (Pereira *et al.*, 2013a).

Translational *SNAP*^{Cd} fusions. To construct a *spoIIQ-SNAP*^{Cd} translational fusion, the *spoIIQ* gene was PCR-amplified using genomic DNA from strain 630Δ*erm* and primers spoIIQ40D and spoIIQR (Table S2) to produce a 1008 bp product. This was inserted between the EcoRI and BamHI sites of pFT58 to create pMS480. We used pMS480, containing the *spoIIQ* gene (see above) and *spoIIQ*-specific primers to convert the histidine codon 120 to a serine codon (found at the homologous position of *spoIIQ* from *Bacillus subtilis*), to produce pMS495. To construct a *spoIIIAH-SNAP*^{Cd} translational fusion, the *spoIIIA* promoter region was amplified using primers spoIIIAAD and spoIIIAA-spoIIIAH, the *spoIIIAH* gene was PCR-amplified using primers P3 and P4 (S2 Table) to produce the 510 bp and 712 bp products, respectively. The *spoIIIA*

promoter was fused to the *spoIIIAH* gene by splicing by overlay extension (SOE) using primers spoIIIAA-spoIIIAH and spoIIIAHR. The PCR product (1222 bp) was inserted between the EcoRI and BamHI sites of pFT58 to create pMS481. To construct a *spoIID-SNAP*^{Cd} translational fusion, the *spoIID* gene was PCR-amplified using genomic DNA from strain 630Δ*erm* and primers spoIID9D and spoIIDR (S2 Table) to produce a 1460 bp product. This was inserted between the EcoRI and BamHI sites of pFT58 to create pMS502. All plasmids bearing *SNAP*^{Cd} fusions were introduced into *E. coli* HB101 (RP4) and then transferred to *C. difficile* 630Δ*erm* and derivatives by conjugation (Heap *et al.*, 2007) (Table 1).

Quantitative analysis of gene expression at the single cell level. For phase contrast and fluorescence microscopy, cells were mounted on 1.7% agarose coated glass slides and observed on a Leica DM6000B microscope equipped with a phase contrast Uplan F1 100x objective and a CCD Ixon^{EM} camera (Andor Technologies) (Serrano *et al.*, 2011). Images were acquired and analysed using the Metamorph software suite version 5.8 (Universal Imaging), and adjusted and cropped using ImageJ (http://rsbweb.nih.gov/ij/). Exposure times were adjusted and defined for each SNAP transcriptional or translational fusion analysed. For quantification of the SNAP-TMR Star signal resulting from transcriptional fusions, 6x6 pixel regions were defined in the desired cell and the average pixel intensity was calculated, and corrected by subtracting the average pixel intensity of the background. Small fluctuations of fluorescence among different fields were corrected by normalizing to the average pixel intensity obtained for the intrinsic autofluorescence of *C. difficile* cells (George *et al.*, 1979).

Statistical analysis. Statistical analysis was carried out using GraphPad Prism (Version 6.0; GraphPad Software Inc.). The non-parametric Kolmogorov-Smirnov test (KS-test) was applied to compare distributions obtained from quantifications of the SNAP-TMR signal. The P-value is indicated for all comparisons whose differences were found to be statistically significant. Although the results presented are from a single experiment, all experiments involving quantification of a fluorescence signal were performed independently three times and only results that were considered statistically significant by a KS-test in all three experiments were considered to be statistically relevant.

Overproduction and purification of SpoIIQ and SpoIIIAH. *C. difficile* 630 *spoIIIAH* (CD1199) and *spoIIQ* (CD0125) coding sequences were PCR-amplified using primer described in Table S2. The resulting fragments were inserted between the NcoI and XhoI sites of pETM-11 (www.embl.de) to create the expression plasmids pPSS002 and pPSS004. The SpoIIQ^{H120S} mutant variant was created by inverse PCR on pPSS002, using primers described in S2 Table to produce pPSS007. *E. coli* Rosetta (DE3) derivatives carrying the desired expression plasmids were grown in LB at 37° C shaking at 180 rpm. Cultures were induced with 1mM IPTG at an OD_{600} of 0.4 - 0.6, and grown for 16h at 30°C before harvesting by centrifugation (4,000x g at 4°C). Cells were re-suspended in 30 ml of 50 mM MES pH 6.0, 500 mM NaCl supplemented with 50 µg/ml lysozyme (Sigma), 20 µg/ml DNase I (Sigma) and 1 EDTA-free Complete protease inhibitor tablet (Roche) and lysed by sonication. After clarification by centrifugation, the cell lysate was filtered through a 0.45 µm filter and loaded onto a 5 ml HisTrap HP (GE Healthcare), primed with 50 mM MES pH 6.0, 500 mM NaCl. After

washing, His_6-tagged protein was eluted in a 0-100% gradient with 50 mM MES pH 6.0, 500 mM NaCl, 500 mM imidazole. The resulting fractions were resolved in 15% SDS-PAGE gels. Fractions containing the desired protein were pooled and protein concentration was determined using a NanoDrop Lite (Thermo) by absorbance at 280nm (A280). The His_6-tag was cleaved by adding 1 mg of TEV protease for every 10 mg of purified protein and the mixture was dialyzed overnight at 4° C against 50 mM MES pH 6.0, 250 mM NaCl. TEV-cleaved protein was separated from uncleaved sample by purification on a 5 ml HisTrap, primed with 50 mM MES pH 6.0, 250 mM NaCl. Unbound protein was collected and concentrated using a 10 kDa MWCO Amicon centrifugal concentrator. Samples were then loaded onto a Superdex 200 PG 26/600 gel filtration column, primed with 50 mM MES pH 6.0, 250 mM NaCl. Fractions were collected, and verified by 15% SDS-PAGE.

Production of an anti-SpoIIIAH polyclonal antibody. Purified SpoIIIAH (as described in the preceding section) was used for the production of a rabbit polyclonal antibody (www.eurogentec.com).

Supporting Results and Discussion

LytM domain structural role

An analysis of the determined structures of BsSpoIIQ-BsSpoIIIAH complex shows that the interface is formed by a 5-strand β-sheet, with 2 strands from SpoIIQ (β2 and β3, Fig. S7C, numbered according to Levdikov and co-authors (Levdikov *et al.*, 2012)) and 3 from SpoIIIAH (β1-β3, Fig.S7C). The interface is further stabilized by α-helices α4 from SpoIIIAH and α1 from SpoIIQ, which stack against the interacting strands (Fig. S7C). Strikingly, according to secondary structure predictions, α1 does not seem to be present in the SpoIIQ protein from *C. difficile* and the whole region is predicted to be more flexible (Fig. 1D and Fig. S7D). In *B. subtilis*, as shown in Fig. S7A and S7B, motif 1 from the LytM catalytic site is found just downstream from SpoIIQ β3 at the complex interface, with the degenerate catalytic site within close proximity of the 5-strand β-sheet. *In vivo,* the proteins are presumed to form large oligomeric rings spanning the inner and outer forespore membranes, with models proposed to contain between 12 (Levdikov *et al.*, 2012) and up to 18 molecules (Meisner *et al.*, 2012) of each protein, embedded in each of the membranes surrounding the forespore. In these assemblies, the LytM regions of neighboring SpoIIQ molecules would lie close to each other, at the multimerisation interface (Fig. S7E). Importantly, in this arrangement, the α1 helix would protrude from one SpoIIQ-SpoIIIAH heterodimer to the next, possibly establishing interactions between units of the multimeric ring. In the absence of this helix, as is predicted to be the case in *C. difficile*, the multimeric interface would require other scaffolding elements. It is possible that coordination of zinc stabilizes this region and allows alternative interactions

between neighboring heterodimers, as well as providing a platform for SpoIIQ-SpoIIIAH complex formation, as discussed in the main text. In the SpoIIQ^{H120S} mutant or when the zinc is chelated away from the protein in our *in vitro* experiments, both the SpoIIQ-SpoIIIAH and the multimeric interfaces would therefore be destabilized due to the lack of those novel structural features.

Consequently, one interesting possibility to consider is that, in *C. difficile* and other *Clostridia* that exhibit intact LytM motifs, Zn^{2+} coordination might play a structural role. The metal could stabilize a flexible region in SpoIIQ in a conformation appropriate for interaction with SpoIIIAH, thereby contributing to the correct assembly of the full multimeric ring. Correct conformation of the individual subunits within the ring and/or ring assembly could then be required for proper function of the SpoIIQ-SpoIIIAH complex. If this is the case, it is the ability to coordinate the metal ion that is important for the function of *C. difficile* SpoIIQ, not a potential enzymatic activity afforded by intact LytM catalytic motifs. However, at this stage, we cannot exclude either option and further work is required to elucidate the role of the LytM domain in the SpoIIQ protein of *C. difficile*.

Supporting Tables

Table S1 - Bacterial strains.

Strain	Relevant Properties*	Origin/Reference
C. difficile		
630Δ*erm*	*C. difficile* 630Δ*erm*	(Hussain *et al.*, 2005)
2649	Δ*erm* Δ*spoIIQ* ^{r}	(Dembek *et al.*, 2015)
AHCD772	Δ*erm* Δ*pyrE*	(Ng *et al.*, 2013)
AHCD721	AHCD718 containing pFT53 (P$_{gpr}$-*SNAP*^{Cd})	This work
AHCD722	AHCD718 containing pFT54 (P$_{spoIIIA}$-*SNAP*^{Cd})	“
AHCD723	AHCD718 containing pFT55 (P$_{sspA}$-*SNAP*^{Cd})	“
AHCD727	630Δ*erm* containing pMS480 (P$_{spoIIQ}$-*SNAP*^{Cd})	“
AHCD728	630Δ*erm* containing pMS481 (P$_{spoIIIA}$-*SNAP*^{Cd})	“
AHCD740	630Δ*erm* containing pMS490 (Split *SNAP*^{Cd})	“
AHCD741	AHCD718 containing pMS481 (*spoIIIAH-SNAP*^{Cd})	“
AHCD742	AHCD718 containing pMS480 (*spoIIQ-SNAP*^{Cd})	“
AHCD768	630Δ*erm* containing pMS495 (P$_{spoIIQ}$- *spoIIQH120S-SNAP*^{Cd})	“
AHCD770	AHCD718 containing pMS495 (P$_{spoIIQ}$-*spoIIQH120S-SNAP*^{Cd})	“
AHCD786	Δ*erm* containing pMS498 (*spoIIQH120S* Split *SNAP*^{Cd})	“
AHCD799	AHCD718 containing pFT69 (P$_{cotE}$-*SNAP*^{Cd})	“
AHCD802	Δ*erm* containing pMS502 (*spoIID-SNAP*^{Cd})	
AHCD803	AHCD718 containing pMS502 (*spoIID-SNAP*^{Cd})	
AHCD812	630Δ*erm* Δ*spoIIIAH*	“
AHCD813	AHCD812 containing pMS502 (*spoIID-SNAP*^{Cd})	
AHCD814	AHCD812 containing pMS480 (*spoIIQ-SNAP*^{Cd})	“
AHCD815	AHCD812 containing pMS481 (*spoIIIAH-SNAP*^{Cd})	“
AHCD816	*sigE*::*intron ermB* containing pMS480 (*spoIIQ-SNAP*^{Cd})	“
AHCD834	AHCD812 containing pFT53 (P$_{gpr}$-*SNAP*^{Cd})	“
AHCD835	AHCD812 containing pFT54 (P$_{spoIIIA}$-*SNAP*^{Cd})	“
AHCD836	AHCD812 containing pFT54 (P$_{sspA}$-*SNAP*^{Cd})	“
AHCD837	AHCD812 containing pFT69 (P$_{cotE}$-*SNAP*^{Cd})	“
E. coli		
PS0050	Rosetta (DE3) (pPSS002); residues 31-222 of SpoIIQ; TEV cleavable N-terminal His$_6$ tag.	“
PS0052	Rosetta (DE3) (pPSS004); residues 29-229 of SpoIIIAH; TEV cleavable N-terminal His$_6$ tag.	“
PS0057	Rosetta (DE3) (pPS007); residues 31-222 of SpoIIQ^{H120S}; TEV cleavable N-terminal His$_6$ tag.	“

*Erm, erythromycin.

Table S2 - Oligonucleotides used in this work.

**Primer**	Sequence (5' → 3')[a]
P1	TTCTTTCTATTCAGCACTGTTATGC
P2	CATCAAGAAGAGCGACTTCG
P3	ATGAAGTTTAATTATAAGGGAAGAGG
P4	AGCTGCGGATCCACCACCACCAAGCTTATTACTATTATTATTTGTAAG
P5	CAATAATTTTATAACATTAACATGG
P6	GTGTTACTTAAAAAATGTAAAT
spoIIQ40D	AAAAGAATTCATGCCGGAACTGTAG
spoIIQR	AGCTGCGGATCCACCACCACCAAGCTTAATTAGACTCATTGGG
spoIIIAAD	TAGATGGTGGAATTCCTAGGGCTTACCAAA
spoIIIAA-spoIIIAH	CCCTTATAATTAAACTTCATCTCTTGTCCTCCTCCTTTG
n*SNAP*^{Cd}R	AAATATGCGGCCGCTTATTGTTGAAATACAGGATGGTGAAGAGC
spoIIIAH-c*SNAP*^{Cd}D	GGTGGATCCGCAGCTGCTGAATCTTTCACCAGACAAGTTTTATGG
c*SNAP*^{Cd}R	CCGCTCGAGTTACCCAAGTCCTGGTTTCCCCAAACG
spoIIQH120SD	GATGTTTGGGAAACTAGCAAAGGTGTAGATATTAG
spoIIQH120SR	CTAATATCTACACCTTTGCTAGTTTCCCAAACATC
spoIIIAH-72D	CCCGGCGCGCCCTATAGCAGGAATATGTGTGG
spoIIIAH-669R	TCCTACTACCACTAACATTGCAG
spoIIAH-1225D	GCAATGTTAGTGGTAGTAGGAGTTGCAGAACAAGCCAATGTG
spoIIIAH-1788R	CCCCCCTGCAGGCCCTCTGACTTTTCAGAGGGC
spoIID-9D	AATGGAATTCGTGTAGTTATAGAAACTG
spoIID-R	AGCTGCGGATCCACCACCACCAAGGTATATATCTTTTATTTTTGTATCTGTG
sQNHisF	GATCGGATCCAAGAAAAAGCTGTTAG
sQFLNHisR	GACCTCGAGTTACTTAATTAGACTCATTGG
sAHNHisF	GATCGGATCCAAGTTTAATTATAAGGGA
SAHNHisR	GATCCTCGAGTTACTTATTACTATTATT
sQH120SF	AGCAAAGGTGTAGATATTAGTTGTACTAAAG
sQH120SR	AGTTTCCCAATCAAGTGTTTTAG

[a] restriction sites are underlined.

Supporting Figures and Figure Legends

Figure S1

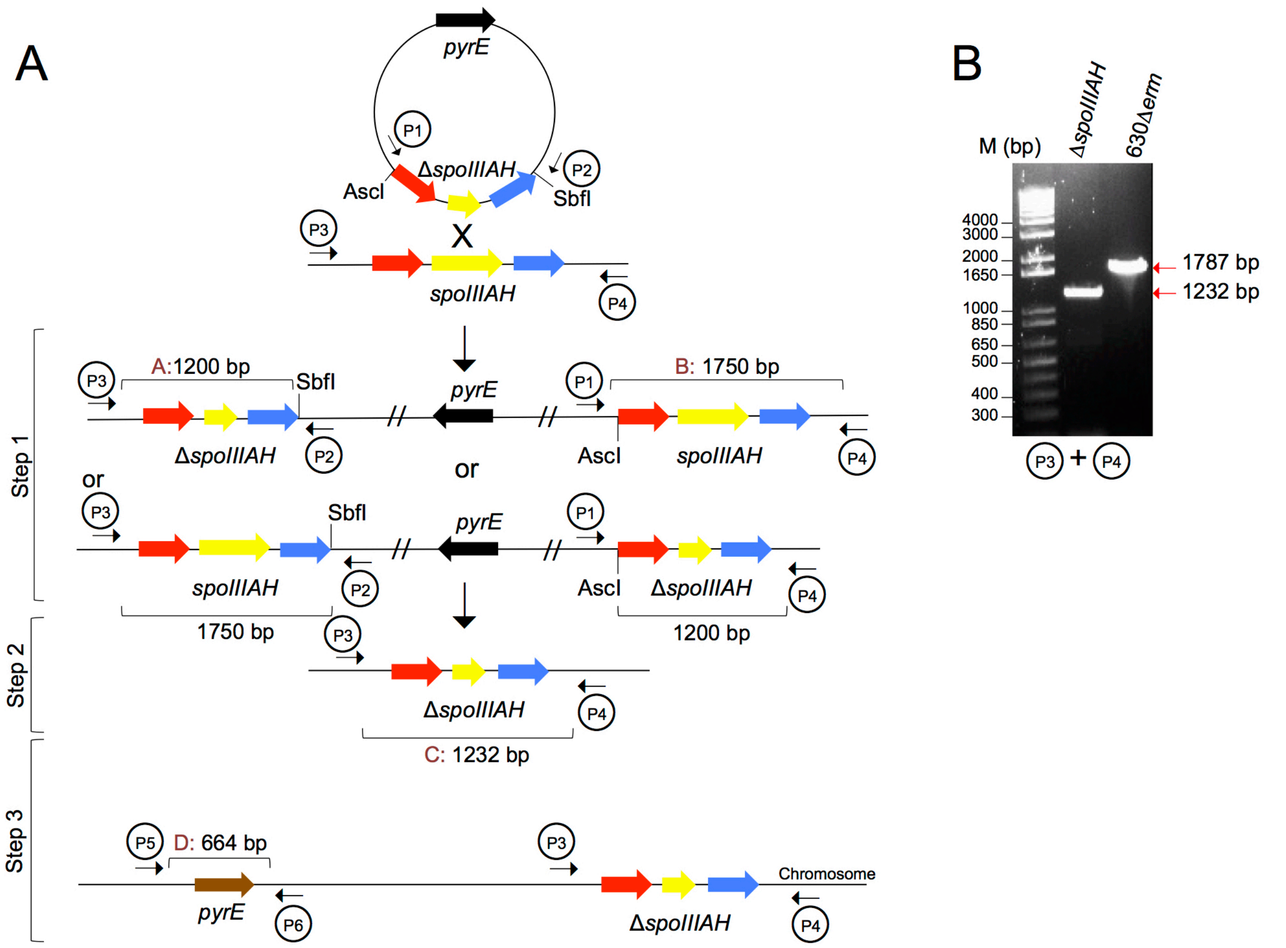

Fig. S1 - Steps in the construction of the *spoIIIAH* in-frame deletion mutant using allele-coupled exchange (ACE). A: PCR fragments A, B, C and D were obtained with primers pairs P2/P3, P1/P4, P3/P4 and P5/P6, respectively. Note that following the initial single reciprocal cross-over, only the integration represented first (of the two possible orientations represented) was obtained. The shorter *spoIIIAH* gene (in yellow) represents the allele with an in-frame deletion of codons 23 to 96 of the 218-codons long open reading frame (see S1 Text for details). **B**: PCR analysis of the parental

630Δ*erm* strain in comparison with the *spoIIIAH* deletion mutant (Δ*spoIIIAH*). The red arrows indicate the products of PCR reactions obtained with chromosomal DNA of the indicated strains with primers P3 and P4 (see panel A). The position on molecular size markers (M, in bp) is shown on the left side of the panel.

Figure S2

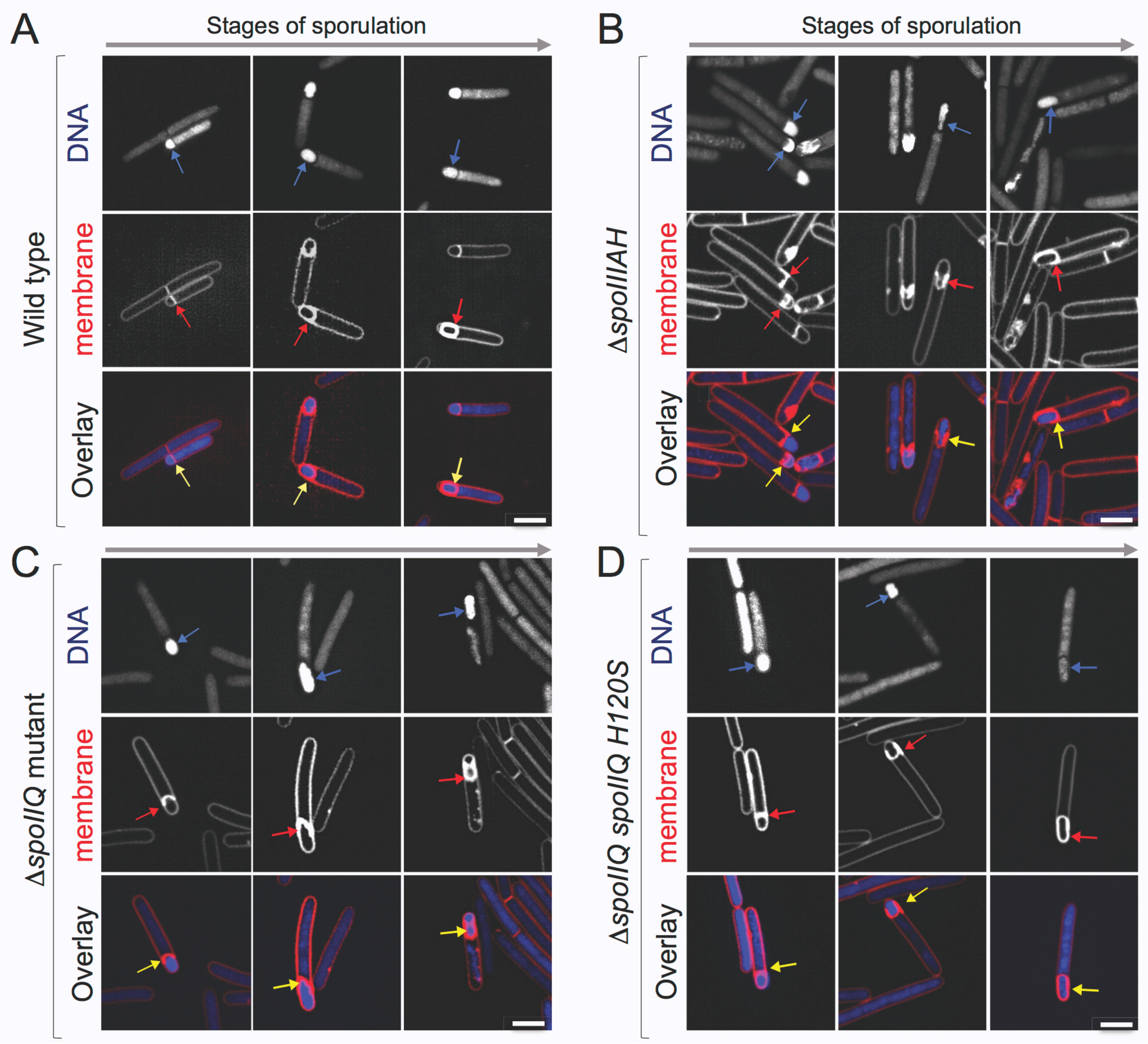

Fig. S2 - Sporulation phenotypes of the *spoIIQ* and *spoIIIAH* mutants. The images show fields of cells for the WT (panel **A**), *spoIIIAH* (**B**), *spoIIQ* (**C**) and *spoIIQ H120S* (**D**) mutants, collected after 14 hours of growth in SM, stained with FM4-64 (membranes, red) and Hoechst (DNA, blue) and imaged by SR-SIM. Blue arrows indicate the condensed forespore chromosome, whereas red arrows indicate the septal membranes, the engulfing membranes and the membranes of the engulfed

forespore. The images are organized according to stages during the engulfment sequence. Note the presence of bulges in the *spoIIQ* and *spoIIIAH* mutants, and the asymmetric movement of the engulfing membranes in some of the *spoIIIAH* sporangia. Scale bar, 2 µm.

Figure S3

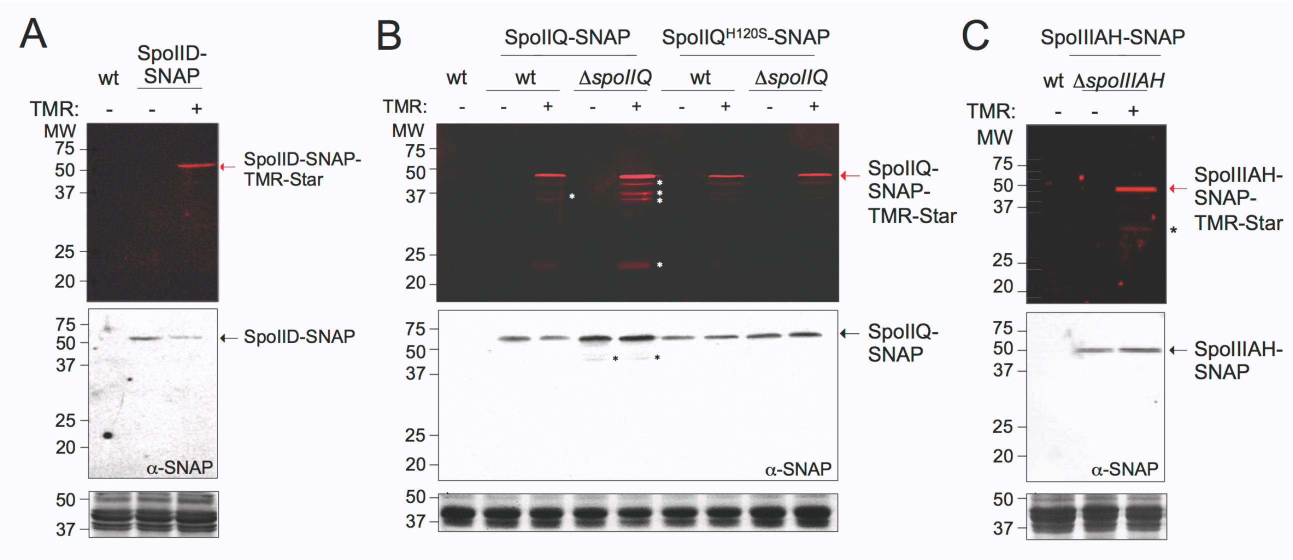

Fig. S3 - Labeling of SpoIIQ-SNAP^{Cd}, SpoIIQ^{H120S}-SNAP^{Cd}, SpoIIIAH-SNAP^{Cd}. Cells from cultures producing SpoIID-SNAP^{Cd} (**A**), SpoIIQ- SNAP^{Cd} or SpoIIQ^{H120S}-SNAP^{Cd} (**B**), or SpoIIIAH- SNAP^{Cd} (**C**) were collected from SM cultures 14 hours after inoculation before ("-" signal) or after ("+") labeling with the TMR-Star substrate and whole cell lysates prepared. Strain 630Δ*erm* (WT, bearing no SNAP^{Cd} fusion) was analyzed in parallel. Proteins in the lysates were electrophoretically resolved (15% SDS-PAGE gels) and visualized by fluoroimaging (top panels) or subject to immunoblot analysis (middle panels) with anti-SNAP antibodies (New England Biolabs). The bottom panels show a section of the same gels used for fluoroimaging, stained with Coomassie, as a loading control. The arrows point to the position of the SNAP^{Cd} fusions. The red arrows point to the position of the TMR-Star-labeled SNAP^{Cd} fusions. The position of molecular mass markers (in kDa) is shown on the left side of the panels.

Figure S4

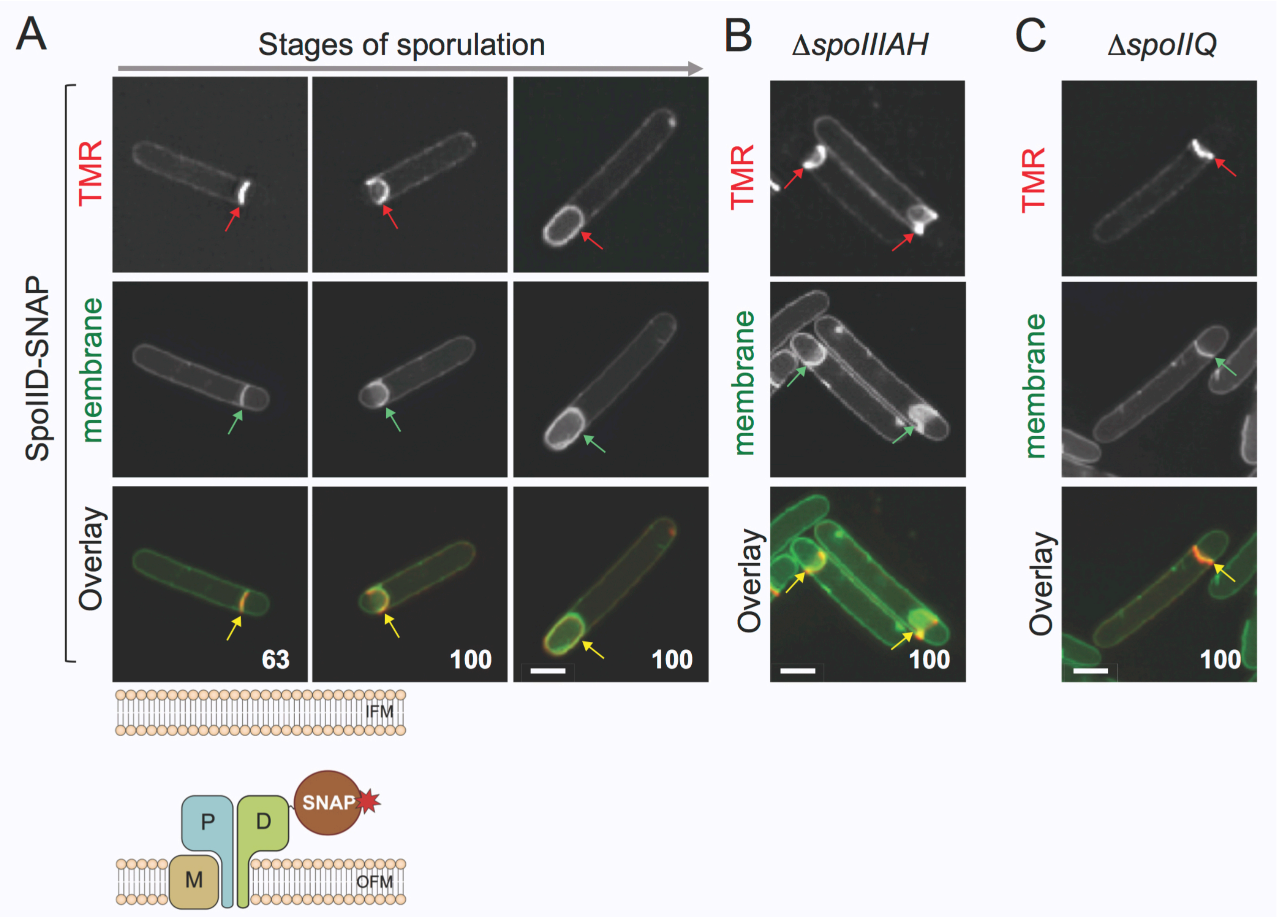

Fig. S4 - Localization of SpoIID-SNAP^{Cd}. The figure illustrates the localization of the DMP component SpoIID-SNAP^{Cd} during different stages of sporulation in a WT background strain (**A**) and in the *spoIIIAH* (**B**) or *spoIIQ* mutants (**C**). Cells were grown for 14 hours in SM and labeled with MTG and TMR-Star prior to imaging by SR-SIM. The signal from the SNAP^{Cd}-TMR-Star complex is indicated by red arrows, whereas the green arrows show the asymmetric septum region, the engulfing membranes, or the membranes of the engulfed forespore, depending on the stage of sporulation illustrated. The numbers represent the percentages of cells showing a similar localization pattern relative to the number of sporulating cells. At least 50 cells were

scored, at each of the represented stages, to derive the indicated percentages. Scale bar, 2 μm. The figure below the panels represents the presumptive topology of the SpoIID (D)-SNAP^{Cd}-TMR-Star labeled protein; the predicted localization of the SpoIIM (M) and SpoIIP (P) proteins, based on the *B. subtilis* model, is also represented.

Figure S5

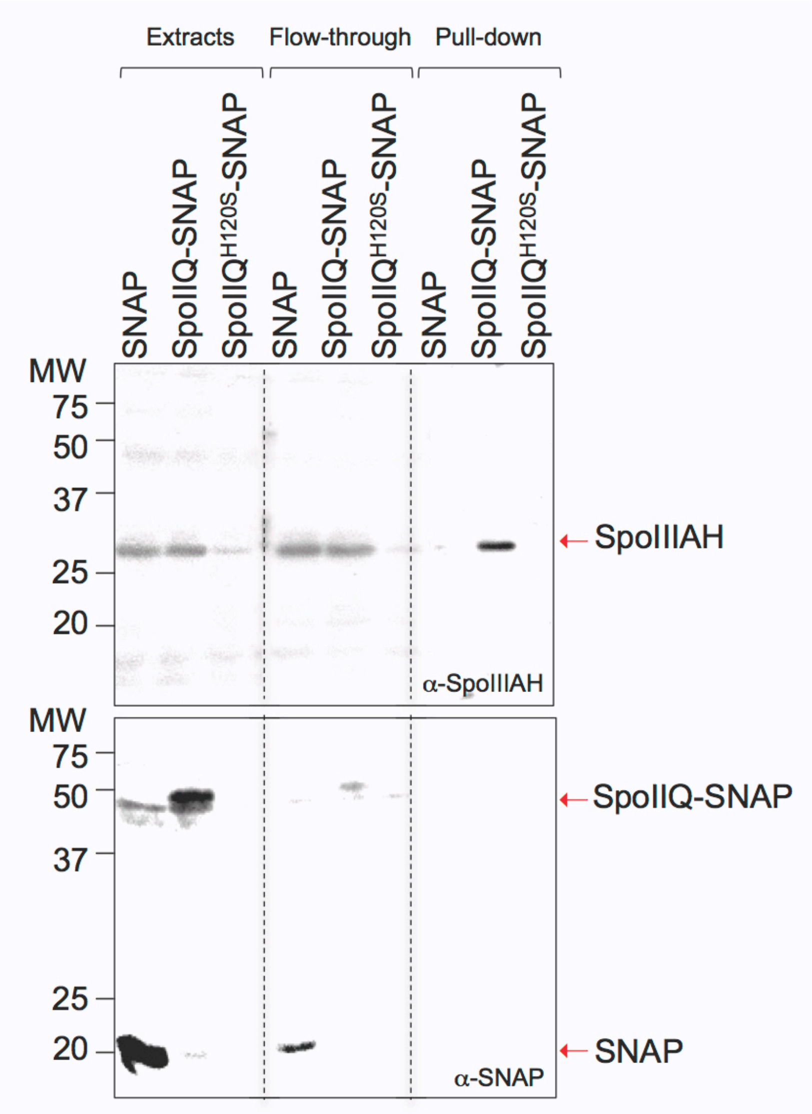

Fig. S5 - SpoIIQ and SpoIIIAH form a complex *in vivo*. Whole cell extracts were prepared from sporulating cells of *C. difficile* producing the SNAP protein under the control of the mother cell-specific *spoIIIA* promoter ($SNAP^{Cd}$), or the $SpoIIQ\text{-}SNAP^{Cd}$ or $SpoIIQ^{H120S}\text{-}SNAP^{Cd}$ fusions under the control of the P_{spoIIQ} promoter, as indicated ("extracts"). The extracts were mixed with SNAP-Capture resin, which covalently binds the SNAP protein. The mixture was washed to remove unbound proteins ("washes")

and proteins interacting with the immobilized SNAP or SNAP fusion were released by boiling ("pull-down"). Proteins in the extracts, flow-through and pull-down samples were resolved by SDS-PAGE and subject to immunoblot analysis with anti-SpoIIIAH (top series of panels) or anti-SNAP (bottom panels) antibodies. Note that SpoIIQ^{H120S}-SNAP^{Cd} is not detected in any of the fractions. Red arrows mark the position of SpoIIIAH, SpoIIQ-SNAP^{Cd} or SNAP. The position of molecular weight markers (MW, in kDa) is shown on the left side of the panels.

Figure S6

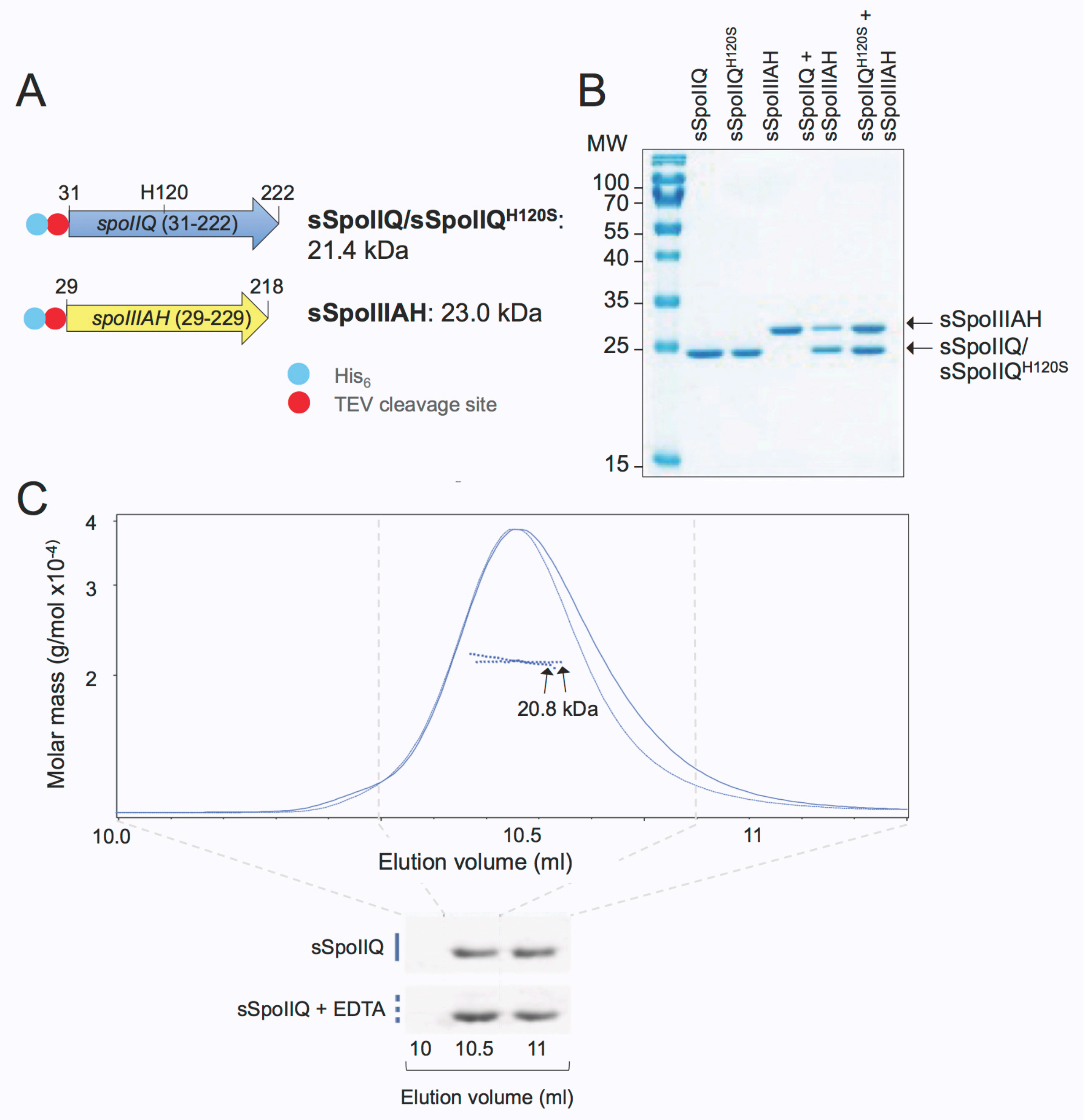

Fig. S6 - Purification of soluble versions of SpoIIIAH, SpoIIQ and SpoIIQ^{H120S}. A: schematic representation of the fusion proteins used for the overproduction and purification of the soluble (prefix “s”) domains of SpoIIIAH, and SpoIIQ/SpoIIQ^{H120S}. Note that the His$_6$ tag (red dot) followed by a TEV protease recognition site (blue dot)

replaces the transmembrane anchor located at the N-terminal end of the native proteins. The expected molecular weight of the proteins, referred to as sSpoIIIAH, sSpoIIQ, or sSpoIIQ^{H120S}, for simplicity, following cleavage by TEV is indicated. **B**: Analysis of purified sSpoIIIAH, sSpoIIQ and sSpoIIQ^{H120S} by SDS-PAGE (first three lanes) reveals that the recombinant proteins exhibit an apparent MW above their predicted mass: sSpoIIQ/ sSpoIIQ^{H120S} is detected as a species of approximately 24kDa, whilst sSpoIIIAH is seen as a band around 30kDa. Importantly, the two individual proteins are detected after pre-incubated sSpoIIQ/sSpoIIQ^{H120S} and sSpoIIIAH (last two lanes). Recombinant proteins were expressed and resolved by SDS-PAGE after Ni^{2+} affinity-chromatography, His$_6$ tag removal, followed by size exclusion chromatography, as detailed in Supplemental Materials and Methods. The position of molecular weight markers (in kDa) is shown on the left side of the panel. **C**: Presence of EDTA does not affect elution profile of sSpoIIQ. SEC-MALLS analysis (top) and corresponding SDS-PAGE gels of purified sSpoIIQ in the absence (blue trace) and presence of 1mM EDTA (dotted blue trace) reveals a similar profile. Fractions (0.5ml) corresponding to elution volumes between 10 and 11ml were analyzed by SDS-PAGE for all chromatography experiments (bottom, dotted lines indicate start and end of collection for each fraction).

Figure S7

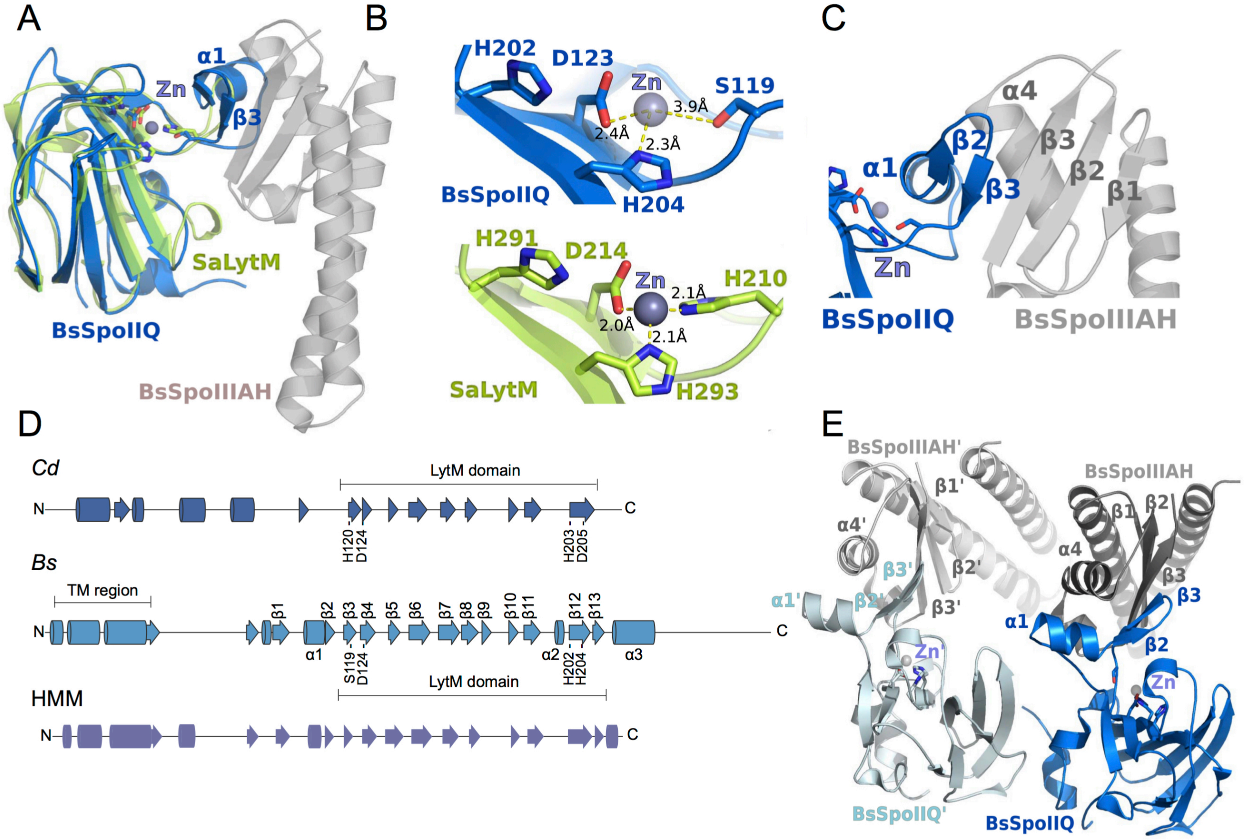

Fig. S7 - Structural role of LytM domains. A: Structural comparison of *B.subtilis* SpoIIQ (BsSpoIIQ, blue) and *S. aureus* active LytM (SaLytM, lime green) shows that SpoIIQ has an overall LytM endopeptidase fold (Firczuk *et al.*, 2005, Meisner *et al.*, 2012, Levdikov *et al.*, 2012). Cartoon representation of BsSpoIIQ (blue):BsSpoIIIAH (white) model (PDB accession code 3TUF, (Levdikov *et al.*, 2012)) superimposed on *S. aureus* LytM (lime green, PDB accession code 2B0P, (Firczuk *et al.*, 2005)). **B**: Zoomed views of *B. subtilis* (top) and *S. aureus* LytM (bottom) domains, with details the residues forming motifs 1 (HxxxD) and 2 (HxH) highlighted. The active site in *S. aureus* with H210, D214 and H293 catalytic residues coordinating the Zn^{2+} ion (sphere) is shown (bottom panel). Using the superimposed protein coordinates, the

position of a potential metal cation in the BsSpoIIQ structure is derived (top panel, transparent sphere). In this scenario, the OH group of S119 would be approximately 3.9Å away from the metal (dotted lines). Thus, coordination of the ion is not favorable in the degenerate LytM motif found in most *Bacilli*, rendering the protein enzymatically inactive. **C**: Detailed view of BsSpoIIQ-BsSpoIIIAH complex interface highlighting the 5-strand β-sheet formed by SpoIIQ β2-β3 and SpoIIIAH β1- β3 strands. The α1 SpoIIQ stacks against the β2 and β3 strands, whilst SpoIIIAH α4 stacks against the remaining 3 strands, stabilizing the interface. The degenerate LytM motif residues (ball-and-stick) and a putative metal ion (transparent sphere) are shown to highlight the proximity to the complex interface. **D**: Secondary structure predictions indicate that SpoIIQ from *C. difficile* (*Cd*, top) has a higher proportion of flexible/disordered regions than *B. subtilis* (*Bs*, middle) or a putative archetypal SpoIIQ (HMM, based on the HMM sequence (Crawshaw *et al.*, 2014). It is particularly noteworthy that regions immediately preceding LytM motif 1 are seemingly unstructured in the clostridial protein. Predictions were carried out with PSIPRED (Buchan *et al.*, 2013). Structural elements are represented as in Fig. 1D. **E**: Cartoon representation of a proposed model of a SpoIIQ-SpoIIIAH dimer. Models for how the heterodimeric SpoIIQ-SpoIIIAH could organize into multimeric rings have been proposed (Levdikov *et al.*, 2012, Meisner *et al.*, 2012). As coordinates of such hypothetical multimers were not available, we replicated the strategy described by Levdikov et al. for their proposed 12mer SpoIIQ-SpoIIIAH ring (Levdikov *et al.*, 2012, Meisner *et al.*, 2012) to analyze potential interactions in more detail. The structural model of BsSpoIIIAH (PDB accession code 3TUF) was superimposed onto a tetramer of EscJ (1YJ7) molecules within the Type III

secretion system ring model (2Y9J). Here, we show two neighboring molecules of the complex: BsSpoIIQ-BsSpoIIIAH (left, blue and dark gray, respectively) and BsSpoIIQ'-BsSpoIIIAH' (left, light blue and light gray, respectively). The key structural elements of the complex interface and the LytM degenerate active site are highlighted as in **C**.

Supporting References

Buchan, D.W., F. Minneci, T.C. Nugent, K. Bryson & D.T. Jones, (2013) Scalable web services for the PSIPRED Protein Analysis Workbench. *Nucleic Acids Res* **41**: W349-357.

Crawshaw, A.D., M. Serrano, W.A. Stanley, A.O. Henriques & P.S. Salgado, (2014) A mother cell-to-forespore channel: current understanding and future challenges. *FEMS Microbiol Lett* **358**: 129-136.

Dembek, M., L. Barquist, C.J. Boinett, A.K. Cain, M. Mayho, T.D. Lawley, N.F. Fairweather & R.P. Fagan, (2015) High-throughput analysis of gene essentiality and sporulation in Clostridium difficile. *MBio* **6**: e02383.

Firczuk, M., A. Mucha & M. Bochtler, (2005) Crystal structures of active LytM. *J Mol Biol* **354**: 578-590.

George, W.L., V.L. Sutter, D. Citron & S.M. Finegold, (1979) Selective and differential medium for isolation of Clostridium difficile. *J Clin Microbiol* **9**: 214-219.

Heap, J.T., O.J. Pennington, S.T. Cartman, G.P. Carter & N.P. Minton, (2007) The ClosTron: a universal gene knock-out system for the genus Clostridium. *J Microbiol Methods* **70**: 452-464.

Hussain, H.A., A.P. Roberts & P. Mullany, (2005) Generation of an erythromycin-sensitive derivative of Clostridium difficile strain 630 (630Deltaerm) and demonstration that the conjugative transposon Tn916DeltaE enters the genome of this strain at multiple sites. *J Med Microbiol* **54**: 137-141.

Levdikov, V.M., E.V. Blagova, A. McFeat, M.J. Fogg, K.S. Wilson & A.J. Wilkinson, (2012) Structure of components of an intercellular channel complex in sporulating Bacillus subtilis. *Proc Natl Acad Sci U S A* **109**: 5441-5445.

Meisner, J., T. Maehigashi, I. Andre, C.M. Dunham & C.P. Moran, Jr., (2012) Structure of the basal components of a bacterial transporter. *Proc Natl Acad Sci U S A* **109**: 5446-5451.

Ng, Y.K., M. Ehsaan, S. Philip, M.M. Collery, C. Janoir, A. Collignon, S.T. Cartman & N.P. Minton, (2013) Expanding the repertoire of gene tools for precise manipulation of the Clostridium difficile genome: allelic exchange using pyrE alleles. *PLoS One* **8**: e56051.

Pereira, F.C., L. Saujet, A.R. Tome, M. Serrano, M. Monot, E. Couture-Tosi, I. Martin-Verstraete, B. Dupuy & A.O. Henriques, (2013a) The spore differentiation pathway in the enteric pathogen Clostridium difficile. *PLoS Genet* **9**: e1003782.

Pereira, F.C., L. Saujet, A.R. Tomé, M. Serrano, M. Monot, E. Couture-Tosi, I. Martin-Verstraete, B. Dupuy & A.O. Henriques, (2013b) The spore differentiation pathway in the enteric pathogen Clostridium difficile. *PLoS Genet*.

Serrano, M., G. Real, J. Santos, J. Carneiro, C.P. Moran, Jr. & A.O. Henriques, (2011) A negative feedback loop that limits the ectopic activation of a cell type-specific sporulation sigma factor of Bacillus subtilis. *PLoS Genet* **7**: e1002220.

Wilson, K.H., M.J. Kennedy & F.R. Fekety, (1982) Use of sodium taurocholate to enhance spore recovery on a medium selective for Clostridium difficile. *J Clin Microbiol* **15**: 443-446.
